# Synthesis, molecular docking studies and biological evaluation of *N*-(4-oxo-2-(trifluoromethyl)-4*H*-chromen-7-yl) benzamides as potential antioxidant, and anticancer agents

**DOI:** 10.1038/s41598-024-59166-5

**Published:** 2024-04-29

**Authors:** Sumalatha Jorepalli, Sreedevi Adikay, Radha Rani Chinthaparthi, Chandra Sekhar Reddy Gangireddy, Janardhan Reddy Koduru, Rama Rao Karri

**Affiliations:** 1 Department of Pharmaceutical Chemistry, Sri Padmavati Mahila Visva Vidyalayam, Tirupati, 517 502 India; 2 Department of Pharmaceutical Chemistry, P.R. Reddy Memorial College of Pharmacy, Kadapa, 516 003 India; 3 Department of Chemistry, Sri Venkateswara College of Engineering, Tirupati, 517 507 India; 4 Department of Environmental Engineering, Kwangwoon University, Seoul, 01897 Republic of Korea; 5 Petroleum and Chemical Engineering, Faculty of Engineering, Universiti Teknologi Brunei, Bandar Seri Begawan, BE1410 Brunei Darussalam

**Keywords:** Chromones, Molecular docking study, Cytotoxic activity, Antioxidant activity, Biophysical chemistry, Chemical libraries, Synthetic biology

## Abstract

A series of novel chromone derivatives of (*N*-(4-oxo-2-(trifluoromethyl)-4*H*-chromen-6-yl) benzamides) were synthesized by treating 7-amino-2-(trifluoromethyl)-4*H*-chromen-4-one with K_2_CO_3_ and/or NaH, suitable alkyl halides and acetonitrile and/or 1,4-dioxane. The obtained products are in high yields (87 to 96%) with various substituents in short reaction times with no more by-products and confirmed by FT-IR, ^1^H, and ^13^C-NMR Spectral data. The in vitro cytotoxic activity was examined against two human cancer cell lines, namely the human lung adenocarcinoma (A-549) and the human breast (MCF-7) cancer cell line. Compound **4h** showed promising cytotoxicity against both cell lines with IC_50_ values of 22.09 and 6.40 ± 0.26 µg/mL respectively, compared to that of the standard drug. We also performed the in vitro antioxidant activity by DPPH radical, hydrogen peroxide, NO scavenging, and total antioxidant capacity (TAC) assay methods, and they showed significant activities. The possible binding interactions of all the synthesized chromone derivatives are also investigated against selective pharmacological targets of human beings, such as HERA protein for cytotoxic activity and Peroxiredoxins (3MNG) for antioxidant activity which showed closer binding free energies than the standard drugs and evidencing the above two types of activities.

## Introduction

A significant class of synthetic and natural compounds with pharmacological activity are chromone derivatives^1–8^. These substances displayed a variety of biological activities, including inhibitors of monoamine oxidase B (MAO-B), anticancer, antimycobacterial, antimicrobial, and inhibitors of the human immunodeficiency virus (HIV-1). These chromone derivatives played a significant role as intermediates in producing dyestuffs, agrochemicals, and pharmaceuticals^3,9,10^. As a result, the topic of study on the synthesis of chromone derivatives is quite interesting and has a lengthy history in the literature^11^. There are reports of certain chromones acting as anti-HIV agents^12^. The chromones klelen and 2,4-thiazolidinedione are utilised as antidiabetic agents to reduce peripheral insulin resistance in individuals with type II diabetes and as antispasmodic agents to treat anginapectoris^13,14^. Substantial anticancer properties were also reported by several phenyl-substituted chromones^15^.

The cyclodehydration of 1-(o-hydroxyaryl)-1,3-diketones or related intermediates, which can be assisted by strong bases or acids (Vilsmeier-Haack reaction), is a standard method for producing chromatones^16^. The Allan-Robinson synthesis, which involves acylation rearrangement and subsequent cyclization, has been widely used to produce them^17^. Additionally, 2-triflouromethylchromone electrophilic nitration was investigated. It has been observed that several lavendustin analogues based on chromone have cytotoxic effects on tumour cell lines^18^. The in vitro cytotoxic activity of novel substituted chromenopyridones was assessed against a range of HCLs, including PC-3 for the prostate, MCF-7 for the breast, IMR-32 for the central nervous system, Hela for the cervix, and Hep-G2 for the liver^19^. Studying cytotoxic effects, particularly on specific cancer cell lines like A-549 (lung cancer) and MCF-7 (breast cancer), serves several important purposes in biomedical research and drug development. These types of cancer are among the most prevalent and deadly worldwide, making them significant targets for drug development studies. Thus, A-549 and MCF-7 cells have become standard models for evaluating cytotoxicity in vitro due to their well-characterized properties and reproducible responses to anticancer agents.

On the other hand, a new class of chemicals known as phosphorus-containing chromone and coumarin derivatives exhibits significant cytotoxicity, alkylating, and anticancer action against specific tumour cell lines^20^. A series of derivatives of 4*H*-chromen-1,2,3,4-tetrahydropyrimidine-5-carboxylate were synthesized and evaluated for their in vitro anti-mycobacterial activity and cytotoxicity against three HCLs, SK-N-SH, A549, and MTB^21^.

**Scheme 1:**
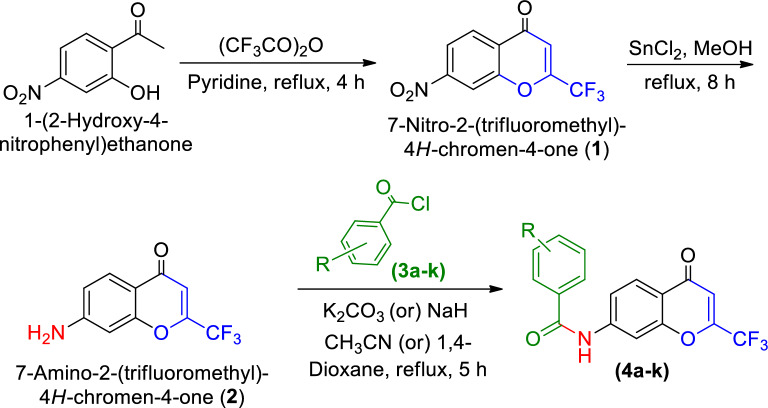
Synthesis of the *N*-(4-oxo-2-(trifluoromethyl)-4*H*-chromen-7-yl) benzamides (**4a-k**).

Nowadays, Cancer is the primary cause of death for humans because of the unchecked, fast multiplication of aberrant cells^22^. Cancer remains a major issue despite significant advancements in its biology and pharmacology; consequently, a safe, effective, and targeted search for novel chemotherapeutic drugs is necessary for their treatment. Our search finds there is no report for the synthesis of *N*-(4-oxo-2-(trifluoromethyl)-4*H*-chromen-7-yl) benzamides and their biological activity. Therefore, herein, we synthesized a series of chromen benzamide derivatives (Scheme 1) and tested their biological activities. Furthermore, integrating in vitro cytotoxicity assays with computational modeling enhances study credibility and translational relevance. Molecular docking predictions correlate with experimental results, validating proposed molecular design strategies. Synthesized chromone derivatives exhibit strong cytotoxic potential, promising as anticancer agents with favorable pharmacological profiles. Supported by robust in vitro and computational evidence, they warrant clinical trial exploration. This research broadens bioactive chromone derivatives and underscores rational drug design's role in advancing novel anticancer therapies, significantly contributing to cancer research.

## Results and discussion

### Chemistry

The *N*-(4-oxo-2-(trifluoromethyl)-4*H*-chromen-7-yl) benzamides (**4a-k**) wαe synthesized by alkylation of 6-amino-2-(trifluoromethyl)-4*H*-chromen-4-one (**3**) with various alkyl/aryl halides in the presence 1.5 mol of K_2_CO_3_ or NaH and 10 mL acetonitrile or 1,4-dioxane stirred at room temperature under nitrogen (Scheme 1). The used amino chromen (**3**) was obtained by reductive amination reaction on the corresponding 6-nitro-2-(trifluoromethyl)-4*H*-chromen-4-one (**2**) in MeOH, with SnCl_2_.2H_2_O at 60 °C for 8 h and this nitro compound was previously synthesized by the nitration of the 2-(trifluoromethyl)-4*H*-chromen-4-one (**1**, 1 g) with nitration mixture in 4 mL of conc. H_2_SO_4_ at 75 °C for 1 h. The intermediate compound (**1**) was obtained by the reaction of 2-hydroxy acetophenone and trifluoroacetic anhydride in the presence of pyridine at 120 °C for 4 h (Table S1).

### Biological evaluation

#### In vitro cytotoxicity by MTT assay

The in vitro anticancer screening of synthesized chromone derivatives **(4a-k)** was tested with MCF-7 and A-549 (these are exhibited good cytotoxic activity on various cell lines such as MCF-7 and A-549.So we have selected these cell lines, based on literature) using the methylthiazolyltetrazolium (MTT) assay method. The reference drug used in this method was Doxorubicin. The IC_50_ values of synthesized compounds against the two cell lines were determined from a graph of cytotoxicity and were shown in Table 1. The graphical representation of IC_50_ values of the title compounds against MCF-7 and A-549 cell lines was demonstrated. From the obtained results, it was observed that all the compounds exhibited significant cytotoxic activity against the A-549 cell line. Among the tested compounds, the compound **4h,** having a *p*-fluorophenyl substitution at chromenbenzamide side chain, displayed high cytotoxicity with an IC_50_ value of 22.09 µg/mL, also followed by the compounds **4b** (with *p*-nitrophenyl), **4e** (with trifluoromethyl), **4c** (with phenyl) and **4k** (with propyl) which exhibited moderate inhibitory potentiality with IC_50_ values of 38.03, 41.99, 44.16 and 48.06 µg/mL respectively.

**Table 1:** In vitro cytotoxic activity of title compounds (**4a-k**) against human lung adenocarcinoma (A-549) and human breast cancer (MCF-7) cell lines.

Compound	IC_50_ (µg/mL)	
	A-549	MCF-7
**4a**	67.36 ± 0.22	12.30 ± 0.12
**4b**	38.03 ± 0.28	9.46 ± 0.72
**4c**	44.16 ± 0.18	9.32 ± 0.16
**4d**	94.98 ± 0.41	12.72 ± 0.42
**4e**	41.99 ± 0.33	10.22 ± 0.46
**4f**	52.3 ± 0.37	9.86 ± 0.40
**4g**	70.36 ± 0.14	13.02 ± 0.60
**4h**	22.09 ± 0.26	6.40 ± 0.26
**4i**	70.18 ± 0.11	11.90 ± 0.48
**4j**	60.22 ± 0.21	11.48 ± 0.36
**4k**	48.06 ± 0.46	10.72 ± 0.64
Doxorubicin	09.18 ± 1.12	15.06 ± 1.08

Similarly, the MCF-7 cell line was much more sensitive to test compounds when compared to that of the A-549 cell line. Most of the test compounds exhibited higher activity levels than the reference compound, Doxorubicin. The cell viability was inhibited significantly in a dose-dependent manner by all the synthesized compounds. When compared to reference drug doxorubicin, the compound **4h** displayed promising cytotoxicity with an IC_50_ value of 6.40 µg/mL, followed by the compounds **4c**, **4b,** and **4f,** which exhibited moderate inhibitory potential with IC_50_ values of 9.32, 9.46, and 9.86 µg/mL respectively against the MCF-7 cell line.

**Figure 1:**
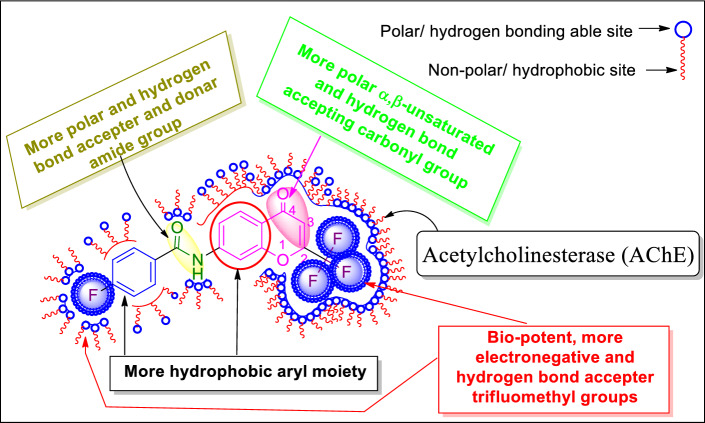
Possible and tentative SAR of compound **4h** as a model for the titled compounds.

As seen in the literature, the 4*H*-chromobenzamide core unit itself has good bioactivity, and in addition, we have also designed and introduced another bio-potent trifluoromethyl group as the best anchoring group in pharmacology. With this, the bioactive potentiality of the core unit was enhanced and showed good to excellent in vitro cytotoxic activity in this work. This bioactivity was tentatively evidenced herein, as shown in Fig. 1.

**Figure 2:**
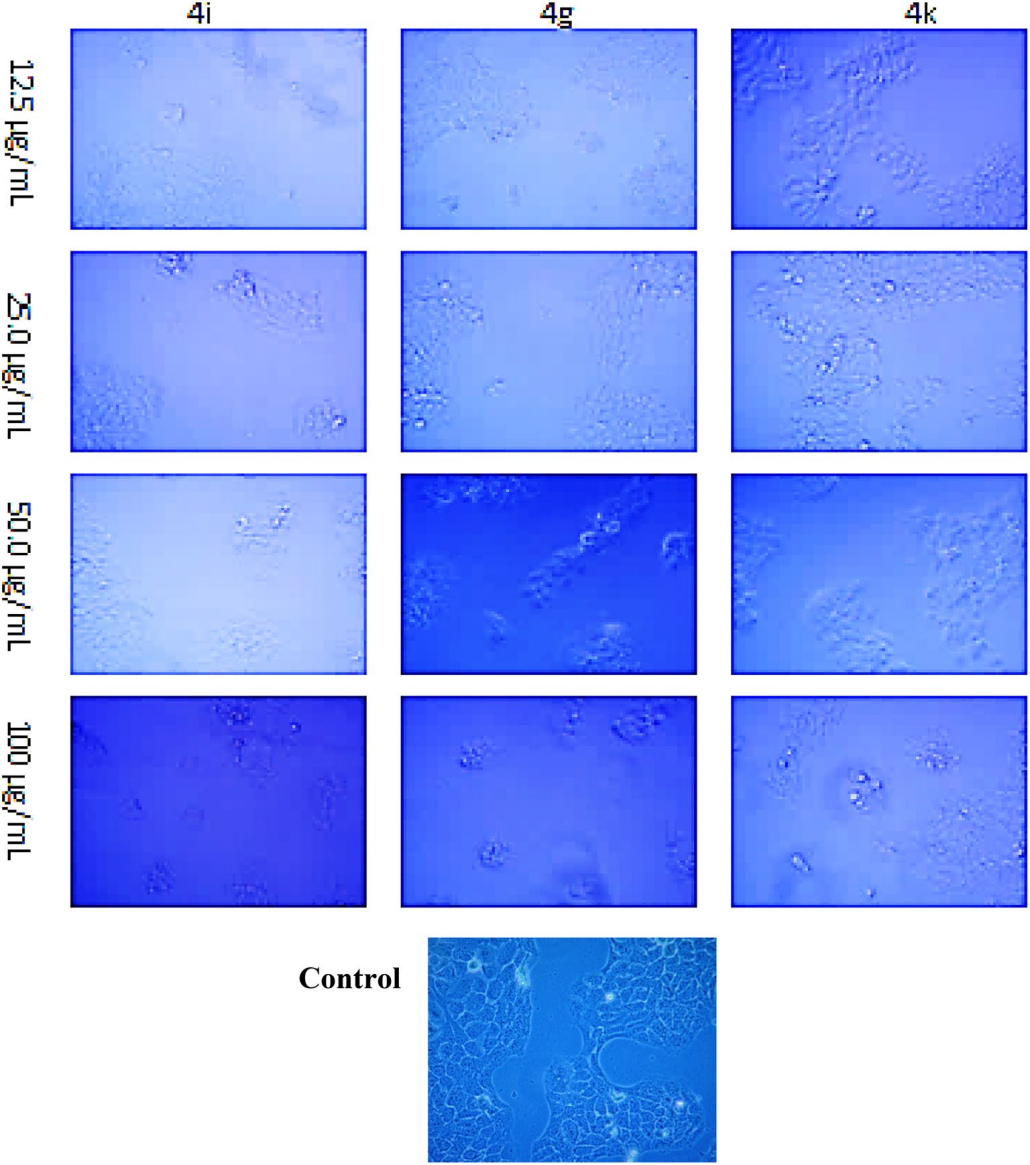
Photomicrographs of human lung adenocarcinoma (A549) cells treated with the test compounds **4h**, **4b**, and **4e** at concentrations of 12.5, 25, 50, and 100 µg/mL after 48 h of drug exposure (magnification 10X).

**Figure 3:**
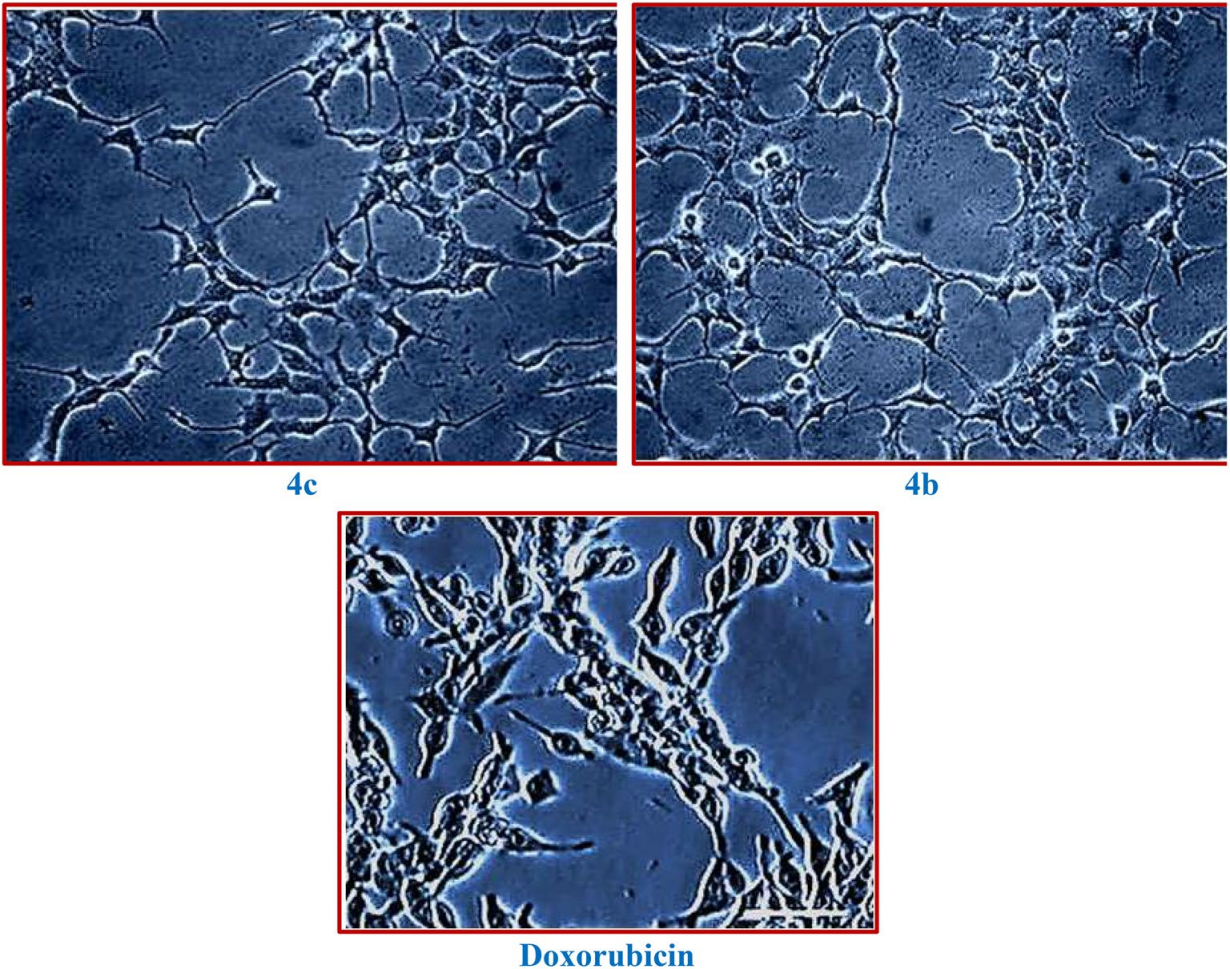
Changes in cell viability during the treatment with title compounds and control in MCF-7 cell lines.

The present results clearly stated that the introduction of electron-withdrawing substitutions, like *p*-fluoro benzene, *p*-nitro benzene, and tri-fluoro methyl groups on the amide side chain of chromenbenzamide at position six are, markedly enhanced the cytotoxicity and while introduction of *p*-toluene sulphonyl, and methane sulphonyl moieties decreased the cytotoxic activity^23,24^. The inverted phase contrast microscope (Primo Vert, Carl Zeiss) was used to observe the cytomorphological abnormalities that occurred to the effect of test compounds for A-549 and MCF-7 cells. The A-549 and MCF-7 cells tested with the synthesized compounds were photographed after 48 h of drug exposure (Figs. 2 and 3). The Results indicated that the control group exhibited typical cobble-stone-like epithelial morphology without any cytological abnormalities. The cells treated with the test compounds showed morphological changes such as membrane blebbing, nuclear disintegration, rounding-off, and membrane degeneration are indicative of cellular apoptosis. Our results of chromone derivatives are further supporting that the findings of cell shrinkage, chromatin condensation, and cellular disintegration in K-562 human myeloid leukaemia cells^25^.

As a result, this investigation identified a novel class of *N*-(4-oxo-2-(trifluoromethyl)-4*H*-chromen-7-yl) benzamides (**4a-k**) with notable target-specific cytotoxicity on human breast cancer (MCF-7) and lung adenocarcinoma (A-549) cell lines. Therefore, most of the synthesized compounds are good therapeutic candidates that will likely be further optimized and developed into anticancer medications in the near future.

#### Antioxidant activity

##### Assay for DPPH radical scavenging activity

Table 2 displays the chemicals' inhibitory effects on the DPPH radical at various doses. The test results were compared with the standard ascorbic acid, and the antioxidant activity was represented in terms of IC50 value (μM), which is the effective concentration for scavenging 50% of the initial DPPH. The results of the titled compounds indicated that compounds **4b (*p***-nitrophenyl)**, 4h (*p***-fluorophenyl), and **4a (***p*-toluene sulfonyl) were displayed high radical scavenging activity among all the compounds with IC_50_ = 38.22 μM, 42.46 μM, 44.40 μM respectively. Compounds **4f** and **4k** exhibited better radical scavenging ability than positive control ascorbic acid. Compounds **4c** and **4e,** with IC_50_values 50.35 ± 0.06 μM, and 52.61 ± 0.27 μM respectively, showed comparable activity when compared to standard ascorbic acid (IC_50_ = 51.06 ± 0.11 μM). As deduced from the IC_50_ data, the chromone derivatives with the lowest anti-radical scavenging capacity were found to be derivatives of **4j** (with IC_50_ = 113.24 μM) followed by **4d** (IC_50_ = 92 μM) and **4i (**IC_50_ = 90.60 μM).

**Table 2:** In-vitro antioxidant activity of the newly synthesized chromone derivatives (**4a-k**) using DPPH free radical scavenging, total antioxidant capacity (TAC) assay, and Hydrogen peroxide and Nitric oxide scavenging methods.

Compound	Antioxidant activity of the title compounds (4a-k) compounds			
	DPPH IC_50_ Conc. (µM) (Mean)	TAC Mean (Ascorbic acid equivalents)	H_2_O_2_ IC_50_ Conc. (µM)^a^	NO IC_50_ Conc. (µM)^a^
**4a**	44.40 ± 0.00	72.84 ± 0.04	45.20 ± 0.08	50.44 ± 0.12
**4b**	38.22 ± 0.04	102.72 ± 0.13	34.71 ± 0.01	41.23 ± 0.17
**4c**	50.35 ± 0.06	61.37 ± 0.37	51.42 ± 0.16	55.07 ± 0.03
**4d**	92.00 ± 0.30	34.76 ± 0.02	79.01 ± 0.02	92.11 ± 0.10
**4e**	52.61 ± 0.27	58.62 ± 0.39	50.16 ± 0.18	39.22 ± 0.07
**4f**	57.59 ± 1.63	34.33 ± 0.00	64.83 ± 0.04	61.62 ± 0.05
**4g**	70.88 ± 2.52	24.47 ± 0.48	71.92 ± 0.11	74.01 ± 0.14
**4h**	42.46 ± 0.49	93.67 ± 0.09	43.11 ± 0.06	38.40 ± 0.02
**4i**	90.60 ± 0.10	41.05 ± 0.27	82.02 ± 0.12	77.28 ± 0.09
**4j**	113.24 ± 22.99	32.92 ± 0.04	93.81 ± 0.01	101.54 ± 0.01
**4k**	61.31 ± 0.46	35.89 ± 0.04	69.10 ± 0.13	62.30 ± 0.16
Ascorbic acid	51.06 ± 0.11	–	52.19 ± 0.05	50.86 ± 0.11

^a^IC_50_ values were calculated by using the data obtained in each test for % scavenging at different concentrations of (25, 50, 75,100 and 125 µM).

The previous studies evidenced that chromone derivatives with 2,3 double bonds, 4-oxo group, and substitutions at position 6 showed good antioxidant activity. The present study results were synchronized with the previous results^26,27^.

##### Total antioxidant capacity assay (TAC)

Table 2 provides the results of the TAC assay. The ascorbic acid equivalents were used to express the antioxidant capabilities. The obtained results of the TAC assay proved a high ascorbic acid equivalent (AAE) for compounds **4b** with *p*-nitrophenyl, **4h** with *p*-fluorophenyl, and **4a** with para toluene sulfonyl substitutions. Hence, they have potent antioxidant activity and can scavenge free radicals efficiently compared to other compounds.

##### Hydrogen peroxide scavenging assay

The synthesized compounds were tested for hydrogen peroxide scavenging activity, and the results were expressed as IC_50_ values in Table 2. Among the tested compounds, the compounds **4b** (*p*-nitrophenyl) and **4h** (*p*-fluorophenyl) displayed high hydrogen peroxide scavenging activity with IC_50_ values 34.71, 43.11 μM respectively, when compared to standard antioxidant ascorbic acid (52.19 μM). The compound **4a** with ***p*-**toluene sulfonyl substitution also showed good activity with a low IC_50_ value of 45.20 μM. Compounds **4c** and **4e** with phenyl trifluoromethyl substitutions exhibited comparable and equipotent activity to standard ascorbic acid with IC_50_ values 51.42 and 50.16 μM, respectively. The results clearly revealed that 2,3 double bonds, 4-oxo group, and the presence of electron-withdrawing substitutions on the amide side chain of chromen benzamide at position 6 enhanced the antioxidant activity^28^.

##### Nitric oxide scavenging activity

Table 2 presents the results of the evaluation of tilted compounds' nitric oxide scavenging ability. The titled compounds **4h, 4e,** and **4b,** possessing *p*-fluorophenyl, trifluoromethyl, and *p*-nitrophenyl substitutions, showed good activity with IC_50_ values 38.40, 39.22, 41.23 μM respectively when compared to standard ascorbic acid (50.86 μM). In comparison to the standard, the other tilting compounds likewise showed moderate to good nitric oxide scavenging efficacy. The current study's findings are consistent with earlier findings^29^.

### Computational studies

#### Molecular properties prediction

Table S2 contains a tabulation of the title compound's computed molecular characteristics. The majority of synthesized derivatives have zero or one violation rate. The obtained results proved that every synthetic derivative exhibited good drug-like properties and complied with Lipinski's criteria. The compounds have good oral bioavailability, as shown by the TPSA of less than 140.

BBB (blood–brain barrier penetration) and the amount of intestinal absorption in humans (HIA%) were projected for each derivative, in addition to the other molecular predictions. All the compounds were found to be well absorbed and to have high HIA% values between 94.54% and 98.58%. Furthermore, it was discovered that they had a moderate BBB-to-CNS penetration rate (0.02–3.63). Therefore, theoretically, all of these parameters showed that the compounds had adequate oral absorption, bioavailability, and reasonable permeability through the blood–brain barrier.

#### Bioactivity score prediction

The synthesized compound's molecular properties prediction is shifted to SI as Table S2.

### Molecular docking studies

#### Cytotoxic activity: molecular docking studies with HERA

**Figure 4:**
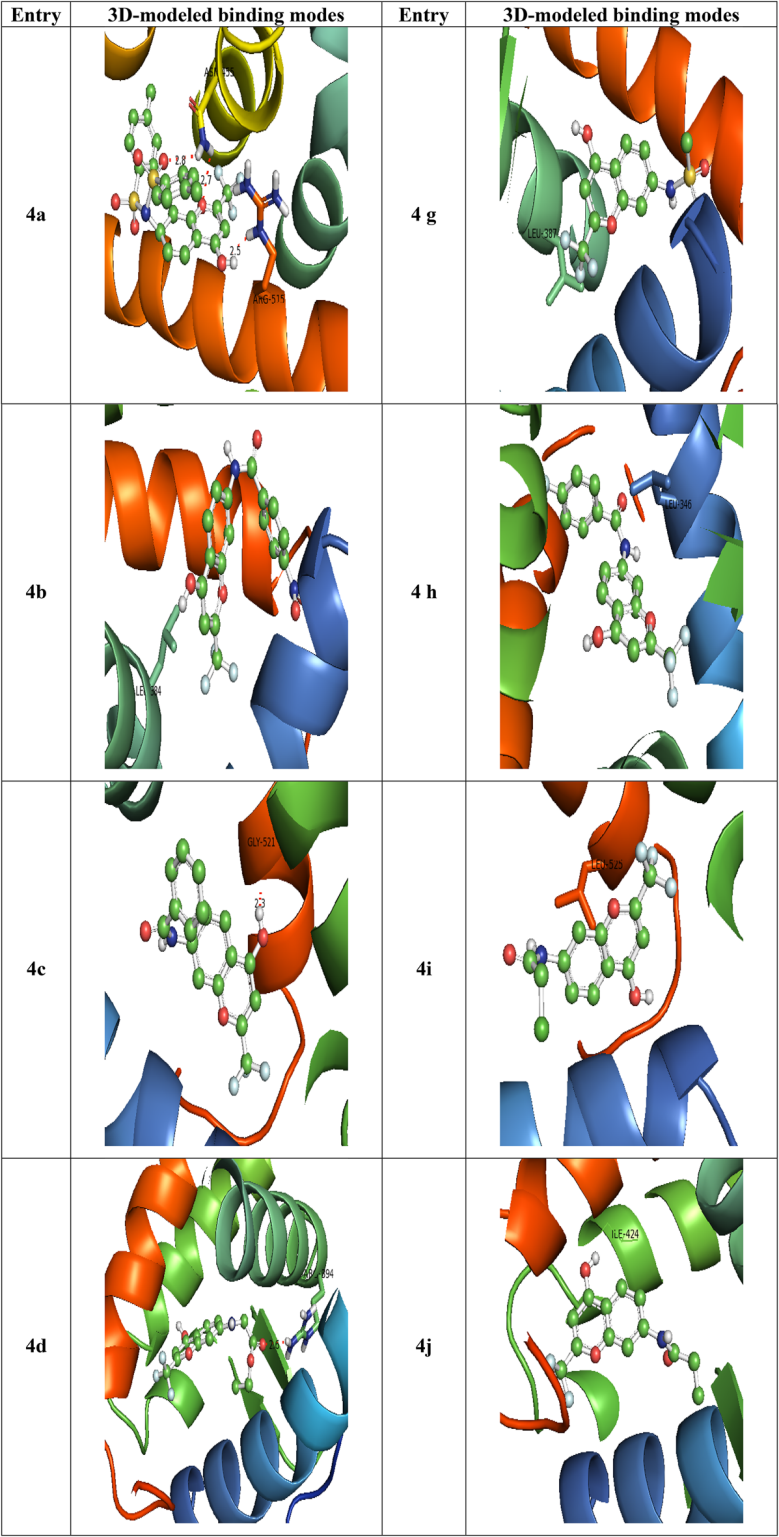

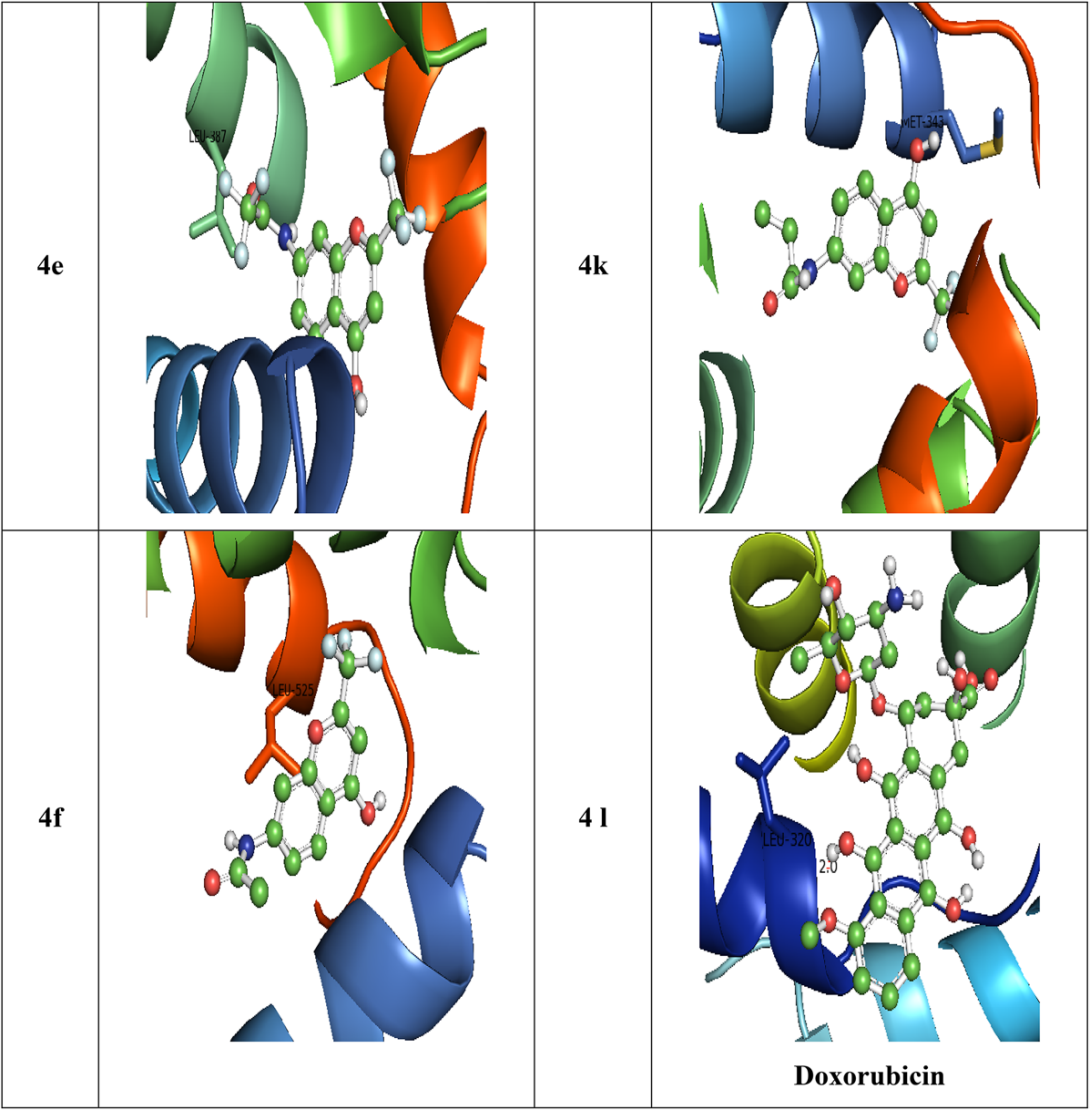
Diagrammatic representation of 3D modelled binding modes of the compounds and standard with the binding domain of Human estrogen receptor alpha protein.

The synthesized chromone derivatives (4a–k) have substantial binding modes against the HERA protein, owing to the docking results. All the drugs, including Doxorubicin (− 6.7 kcal/mol), had dock scores that were higher than those of the reference chemical. The H-bonds, binding affinities, and energy profiles of compounds (4a-k) and Doxorubicin concerning the amino acids in the enzyme's active site are shown in Table 3. Figures 4 and S1 depict the best lead compounds' 3D and 2D modelled interactions with the HERA protein. The synthesized compounds have binding free energy in the range of − 9.6 to − 7.5 kcal/mol, whereas the standard drug Doxorubicin showed a score of − 6.7 kcal/mol. Compound **4h** *(p*-fluorophenyl) has the best binding energy of − 9.6 kcal/mol and showed the highest affinity among all the synthesized compounds. Compound **4h** with *p*-fluorophenyl substitution fits into the binding cleft of HERA protein and forms H-bonding between carbonyl group with side chain amino acids, Leu 346 with bond length 3.1 Å (Fig. 4). The compounds **4b** with p-nitrophenyl and **4c** with phenyl substitutions form H-bonding (binding energy of − 9.0 kcal/mol) between hydroxyl group with side chain amino acids Leu 384, Gly 521 of HERA protein with bond lengths 2.6, 2.3 Å respectively. The compounds **4e (**trifluoromethyl)**, 4k** (propyl), **4i** (chloromethyl), and **4j** (ethyl) also showed good binding affinities in the range of − 8.4, − 8.2, − 7.8, − 7.8 kcal/mol respectively (Fig. 4). All the synthesized compounds have shown hydrophobic interactions against HERA protein except **4a** (*p*-toluene sulphonyl), **4c** (phenyl), and **4d** (ethoxy).

**Table 3:** Bonding characterization of synthesized compounds (**4a-k)** and doxorubicin (Reference compound) against human estrogen receptor alpha protein.

Compound	B E (kcal/mol)	Bonding interaction	Bond length (Å)	Bond angle (°)	Bond type
**4a**	− 7.6	Arg 515 CB ….OH	2.5	88.1	H-acc
		Asn 455 CA ….HN	2.7	96.0	H-don
		Asn 455 CA ….HN	2.5	80.7	H-don
**4b**	− 9.0	Leu 384 CZ …HO	2.6	138.8	H-don
**4c**	− 9.0	Gly 521 CZ ….HO	2.3	91.4	H-don
**4d**	− 7.5	Arg 394 CZ ….OC	2.6	100.1	H-acc
**4e**	− 8.4	Leu 387 CZ ….HO	2.6	100.8	H-don
**4f**	− 7.7	Leu 525 CB ….CO	2.8	120.0	H-acc
**4g**	− 7.5	Leu 387 CZ ….CF	2.6	109.7	H-acc
**4h**	− 9.6	Leu 346 CZ ….OC	3.1	118.3	H-acc
**4i**	− 7.8	Leu 525 CB ….OC	3.1	130.5	H-acc
**4j**	− 7.8	Ile 424 CB ….HO	3.7	90.3	H-don
**4k**	− 8.2	Met 343 CZ ….HO	2.0	97.0	H-don
Doxorubicin	− 6.7	Leu 320 CZ ….HO	2.0	122.7	H-don

The best binding affinity (ΔG, − 9.6 kcal/mol) towards HERA protein was displayed by the compound **4h,** which exhibited marked in vitro cytotoxicity on both cell lines A549 and MCF-7 and this active compound **4h** was found to establish six types of interactions: (i) halogen(fluorine): with LEU:387, GLU:353, (ii) Pi-sigma: with LEU:346, (iii) Pi-sulfur: with MET:421, (iv) Pi-Pi-T-shaped: with PHE:404, (v) alkyl: with ALA:350, (vi) pi-alkyl: with LEU:391 (Figure S1).

From the results of docking studies, it was found that the newly synthesized chromone-linked amide derivatives have a good binding affinity with HERA and confirmed the in vitro cytotoxicity in ERα positive cell line MCF-7 and A549. Present study results also support the previous results^30,31^. As a result, these interactions may provide supporting evidence for in vitro cytotoxic screening of the compounds on various cell lines.

#### Antioxidant activity: molecular docking studies with Peroxiredoxins (PDB: 3MNG)

The human 3MNG protein is a good target for antioxidant activity, and therefore, the synthetic compounds 4a–k was subjected to a selective pharmacological target for their molecular docking investigation. The compounds' docking results against the 3MNG protein showed that when compared to the control drugs tocopherol (− 5.0 kcal/mol) and ascorbic acid (− 5.4 kcal/mol), respectively, all the compounds in the title had significant binding modes, with dock scores ranging from − 5.9 to − 7.4 kcal/mol. The H-bonds, binding affinities, and energy profiles of compounds 4a-k and reference drugs towards the enzyme's active site amino acids are shown in Table 4. Figures S1 and 5 exhibit the compounds' 3D and 2D binding modes. The compounds **4b (***p*-nitrophenyl), **4h** (*p*-fluorophenyl), **4a** (*p*-toluene sulphonyl), and **4c** (phenyl) have formed high dock with the target protein 3MNG, followed by the rest of the compounds. Out of all the synthesized compounds, **4b** with *p*-nitrophenyl substitution showed the best binding affinity (− 7.4 kcal/mol) with the 3MNG protein when compared to standard drugs. Compound **4b** fits into the binding cleft of 3MNG protein and forms an H-bonding between hydroxyl, a nitro group with side chain amino acids As 99, Ala 78 with bond length 2.3, 2.0 Å (Figure S2). Out of all of them, 4c and 4 k (propyl) have bonded hydrophobically to the 3MNG target protein.

**Table 4:** Bonding characterization of standards and synthesized compounds (**4a-k**) against 3MNG protein.

Compound	B E (kcal/mol)	Binding interaction	Bond length (Å)	Bond angle (^o^)	Bond type
Tocopherol	− 5.0	Val 94 CB ….HO	1.9	115.7	H- don
Ascorbic acid	− 5.4	Ala 90 CZ ….OC	2.8	120.8	H- acc
		Glu 91 CZ ….HO	2.7	118.0	H- don
		Gly 92 CZ ….OH	2.3	100.1	H- acc
**4a**	− 6.6	Val 94 CB ….OC	2.3	121.8	H- acc
**4b**	− 7.4	As 99 CZ ….HO	2.3	70.8	H- don
		Ala 78 CZ ….ON	2.0	81.9	H- acc
**4c**	− 6.4	Glu 16 CB ….HN	2.1	88.5	H- don
**4d**	− 5.9	Asn 2 CZ ….OH	2.3	91.7	H- acc
		Arg 86 CB ….OC	2.3	103.0	H- acc
**4e**	− 6.1	Glu 16 CB ….HN	2.4	127.7	H- don
		Arg 86 CA ….OC	2.8	133.8	H- acc
**4f**	− 6.1	Glu 16 CB ….HN	2.4	127.7	H- don
		Arg 86 CA ….OC	2.8	133.8	H- acc
**4g**	− 6.0	Gly 92CB ….HO	2.2	84.7	H- don
		Asn 21 CB ….OS	2.5	120.9	H- acc
**4h**	− 6.9	Gly 92 CB ….OC	2.2	138.1	H- acc
**4i**	− 5.9	Glu 16 CB ….HN	2.4	127.7	H- don
		Arg 86 CA ….OC	2.8	133.8	H- acc
**4j**	− 5.9	Asn 21 CB ….OC	2.6	123.7	H- acc
**4k**	− 6.0	Asn 21 CB ….OC	2.8	114.0	H- acc

**Figure 5:**
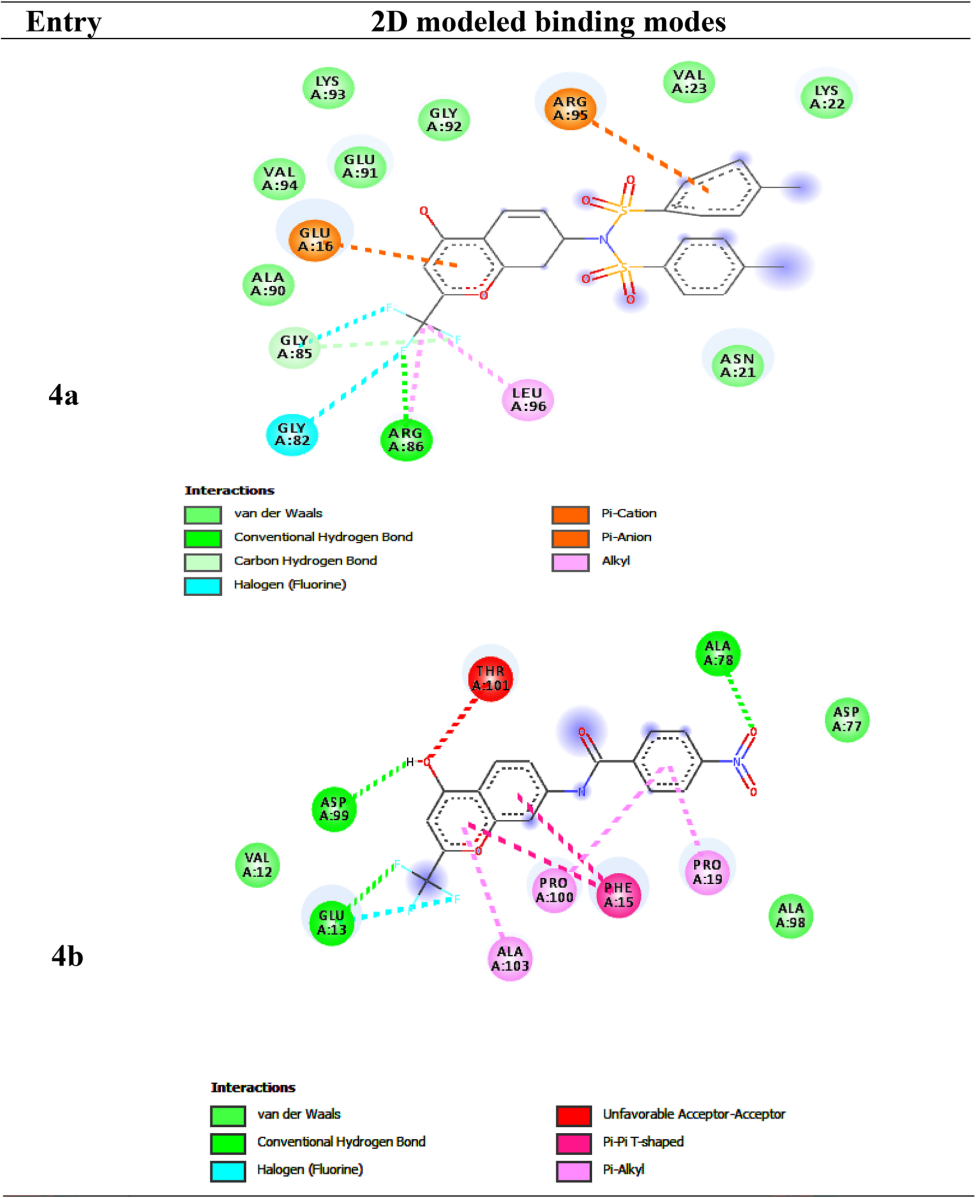
2D binding domains of lead compounds against 3MNG protein.

The compound **4b** exhibited high binding affinity (− 7.4 kcal/mol) towards 3MNG protein and displayed potential *invitro* antioxidant activity. The active compound **4b** was found to establish four types of interactions: (i) halogen (fluorine) with GLU:13, (ii) unfavourable acceptor-acceptor: with THR:101, (iii) Pi-Pi-T-shaped: with PHE:15, (iv) Pi-alkyl: with PRO:19, PRO:100, ALA:103 (Fig. 5).

From the results, it was clear that the presence of *p*-nitrophenyl substitutions containing chromone-linked amides was important for the antioxidant activity. The synthetic compounds' binding modes showed that they fit into the 3MNG protein's binding pocket more stably. The results of the present study were consistent with those from earlier studies^31^.

In this present study, chromone derivatives of *N*-(4-oxo-2-(trifluoromethyl)-4*H*-chromen-7-yl) benzamides (**4a-k**) were synthesized by short reaction times with high yields. The chromone derivatives showed good in vitro cytotoxicity and antioxidant activity. Upon in silico studies, titled compounds displayed good binding interactions with the target proteins (HERA and 3MNG). Thus, synthesized compounds might be useful cytotoxic and antioxidant drug candidates with good oral bioavailability.

## Experimental section

### Chemistry

HCl (7647-01-0), CH_3_COOC_2_H_5_ (141-78-6), CH_2_Cl_2_(75-09-2) are acquired from Sd fine, India, 2-Hydroxy acetophenone (118-93-4), Na_2_SO_4_(239,313), H_2_SO_4_ (7664-93-9), Hexane (110-54-3), HNO_3_(7697-37-2), are purchased from Mark India, and L-Glutamine–(J60573.A1), Streptomycin (15,465,739), are purchased from Alfa Aesar. The given melting points were calculated in open capillaries using the Stuart melting point equipment at °C and are uncorrected. The UV spectra were recorded using a Systronics UV–visible spectrometer, while the IR spectra were recorded in cm^−1^ using a Perkin-Elmer Spectrum BX-I Infrared Spectrophotometer utilizing the KBr pellet. The Jeol JNM-ECS400 model instrument is used to capture the NMR data at 400 MHz, with TMS serving as an external reference. Iodine vapour is utilized to see the sample spots on the Silica gel-G coated glass plates, which are used to verify the purity of the compounds mentioned.

#### Synthesis of 2-(trifluoromethyl)-4H-chromen-4-one (1)

2-Hydroxy acetophenone (1 eq.) was dissolved in trifluoroacetic anhydride (1.2 eq.), and pyridine (2 eq.) was added to the reaction mixture. The mixture was heated at 120 °C for **4h**. After completion of the reaction, judged by TLC, the reaction mixture was treated with 1 M HCl, followed by water wash, and then the unreacted 2-hydroxy acetophenone was removed by washing with 1 M NaOH. After drying the obtained organic phase with anhydrous Na_2_SO_4_, the solvent was removed in a rotary evaporator to yield the crude compound, which was then pure 2-(trifluoro methyl)-4H-chromen-4-one (1). Using a 5% ethyl acetate/hexane eluent system, column chromatography was used to purify the resultant compound.

#### Synthesis of 7-nitro-2-(trifluoromethyl)-4H-chromen-4-one (2)

A chromone (1, 1 g) solution in 4 mL of concentrated H_2_SO_4_ was combined with 1 mL of conc. H_2_SO_4_ and 1 mL of 70% HNO_3_. The reaction mixture was cooled after one hour of heating at 75 °C and then poured over crushed ice while being stirred. When the precipitate was filtered, rinsed with water, and dried, it yielded the pure nitro compound 6-nitro-2-(trifluoromethyl)-4H-chromen-4-one (2). The obtained organic phase was dried with anhydrous Na_2_SO_4_, and the solvent was removed in a rotary evaporator to provide the crude compound of 2-(trifluoro methyl)-4H-chromen-4-one (1). Column chromatography with a 5% ethyl acetate/hexane eluent system was used to purify the resulting compound.

#### Synthesis of 7-amino-2-(trifluoromethyl)-4H-chromen-4-one (3)

After the reaction is completed, the solvent is removed in a rotary evaporator and diluted with C_2_H_5_COOCH_3_ before being quenched with NaHCO_3_. The resulting slurry was filtered through a celite bed, the organic layer was dried with anhydrous Na_2_SO_4,_ and the solvent was removed in a rotary evaporator to yield the amine compound 6-amino-2-(trifluoromethyl)-4H-chromen-4-one (3).

#### General procedures for the synthesis of titled amides (4a-k)

In a round bottom flask, the following were added: 1 mol of an appropriate amine; 1.5 mol of K_2_CO_3_ (for 4a–4c synthesis) or NaH (for 4d–4 k synthesis); 1 mol of appropriate alkyl halides; and 10 mL of either acetonitrile (for 4a–4c synthesis) or 1,4–dioxane (for 4d–4 k synthesis). The mixture was then agitated at room temperature while being exposed to a nitrogen atmosphere. The reaction mixture was dried off under low pressure once the reaction was finished, which was observed by TLC. After dissolving the obtained compound in CH_2_Cl_2_, it was twice washed in water using a separating funnel. After repeatedly washing the last aqueous layer with new CH_2_Cl_2_, the collected organic fractions were mixed and dried over anhydrous Na_2_SO_4_. The solvent was then extracted under low pressure, yielding the crude as well as the titled compounds. Which, after being purified by column chromatography, yields huge quantities of the desired compounds (4a-k).

**Scheme 2:**
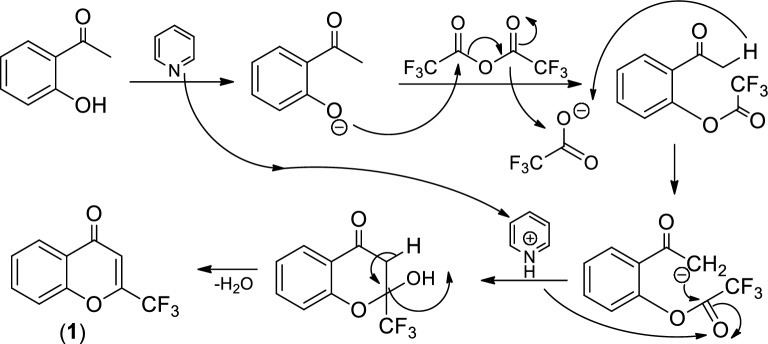
Pictorial representation of the simple condensation reaction of chromone derivative (**1**).

In this synthesis, the titled products are obtained by simple condensation and nitration, followed by the reduction of the nitro group. At first, the simple condensation reaction of *o*-hydroxy acetophenone was reacted with pyridine, and then the phenolic group underwent condensation with trifluoroacetic anhydride followed by the cyclisation of obtained intermediate (Scheme 2), resulting in 2-(trifluoromethyl)-4*H*-chromen-4-one (**1**).

In these reaction conditions, products are observed in high yields irrespective of the nature of the alkyl/aryl halides (Table 1).

#### Physical and spectral data of representative compounds, 4-methyl-N-(4-oxo-2-(trifluoromethyl)-4H-chromen-7-yl)-N-tosylbenzenesul fonamide (4a)

Yield: 95%; IR (KBr) *ν*_*max*_: 3078.8, 3030.3 (C=C_Stretch_), 2926.2 & 2855.7 (C–H_Stretch_), 1675.9 (C=O), 1602.4 (C–), 1476.1 & 1449.0 (C–H_Bend_), 1380.1 (S=O_Stretch_) cm^−1^; ^1^H NMR (CDCl_3_) *δ*: 7.87 (4H, *d*, ^*3*^*J*_*H-H*_ = 8.0 Hz, Ar–H_(14,14´,18&18´)_), 7.78 (4H, *d*, ^*3*^*J*_*H-H*_ = 8.0 Hz, Ar–H_(15,15´,17&17´)_), 7.58–7.56 (1H, *d*, ^*3*^*J*_*H-H*_ = 10.0 Hz, Ar–H_(5)_), 7.54 (1H, *s*, = CH_(3)_), 6.85–6.76 (2H, *m*, Ar–H_(6&8)_); ^13^C NMR (CDCl_3_) *δ*: 177.1 (C-4), 160.4 (C-2), 149.2 (C-9), 145.3 (C-7), 124.8 (C-16&16´), 122.5 (C-13&13´), 119.4 (C-5), 109.4 (C-15,15´,17&17´), 103.6 (C-14,14´,18&18´), 98.9 (–CF_3_), 95.1 (C-8), 61.6 (C-3), 14.1 (–CH_3_); Mass [M + Na]^+•^ = 560, [M + 1]^+•^ = 537; Anal. Calcd. for C_24_H_18_F_3_NO_6_S_2_: C, 53.63; H, 3.38; N, 2.61; found: C, 53.60; H, 3.37; N, 2.60.

All the other compound’s physical and spectral data associated with this article are given as supplementary information.
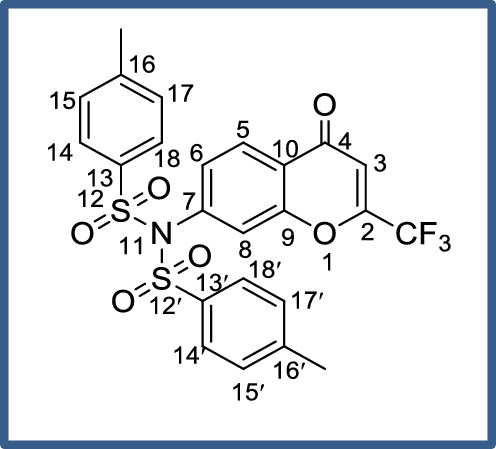


### In vitro cytotoxicity studies

#### Maintenance of cell lines

The King Institute of Preventive Medicine in Chennai provided A549 and MCF-7 cells. The cells were cultured in a T25 mL vented flask with MEM supplemented with 10% foetal bovine serum, 3% L-Glutamine, Streptomycin (100 g/mL), Penicillin (100 IU/mL), and Amphotericin B along with 7.5% Na_H_CO_3_, and the flask was incubated at 37 °C in a 5% CO_2_ incubator. An inverted microscope confirmed the formation of an approximately 80–90% confluent monolayer (adherent) after 3 days. It was then subcultured and used for further research using a TPVG solution and a small amount of media.

#### Cytotoxicity assessment using methyl thiazolyltetarazolium (MTT) assay

The cytotoxicity of the drugs on A549 and MCF-7 cells was assessed by MTT assay^32^. After the cells were gathered and seeded on 96-well plates, they were incubated for 2**4h** at 37 °C with 5% CO_2_ to promote cell attachment. The mixture was incubated for 48 h with varying concentrations of the test compounds after 12 h. Following incubation, phenol red and FBS-free media were added to each well, along with 15 mL of MTT (5 mg/mL) dye, and the plate was covered with Al foil and incubated for another **4h**. The medium was aspirated after incubation, and each well received 100 L of DMSO to dissolve the formazan crystals^33^. At 570 nm, the optical density (OD) was measured. The percentage of cell inhibition was calculated using the formula below.

$$\%\, of\, cellviability= \frac{OD\, of\, test}{OD\, of\, control} \times 100$$

## In vitro antioxidant studies

### Antioxidant activity by 2,2-diphenyl-1-picrylhydrazyl (DPPH) scavenging assay

The ability to scavenge the stable free radical DPPH was measured using a modified method^34^. Test compounds (50 L) at various concentrations (25, 50, 75, 100, and 125 M) were combined with 200 L of methanolic solution containing 100 M DPPH radical. The mixture was thoroughly mixed and left to stand in the dark for 10 min (until stable absorption readings were achieved). The absorbance at 517 nm was used to calculate the DPPH radical's decrease. The radical scavenging activity (In h%) was calculated as a % of DPPH discolouration using the following equation.

$$\mathrm{I\% }= \left[\frac{({A}_{Control}-{A}_{Sample})}{{A}_{Control}}\right] \times 100$$

The graph of the inhibition percentage against test concentration was used to determine the test compound concentration that provided 50% inhibition (IC_50_ of DPPH), with vitamin C serving as the standard. There were three duplicates of each test run.

#### Total antioxidant capacity assay

This assay is based on the reduction of Mo (VI) to Mo(V) in the sample and the subsequent formation of a green phosphate/Mo(V) complex at an acidic pH. In test tubes containing 1 mL of test solutions (50 M), 1 mL of Mo(V) reagent solution (0.6 M H_2_SO_4_, 28 mM Na PO_4_, and 4 mM (HH_4_)_6_ Mo_7_O_24_) was added. Vortexed tubes were incubated at 90 °C for 90 min. After allowing the tubes to cool to room temperature, the absorbance of the samples was measured at 695 nm. The results are given in milligrams of ascorbic acid equivalent per gram of test compound^35^.

#### Hydrogen peroxide scavenging method

The modified approach was used for this method^36^. A solution of H_2_O_2_ (40 mM) was prepared in PO_4_^−3^ buffer (pH 7.4). At concentrations of 25, 50, 75, and 100 M, the test compounds were added to an H_2_O_2_ solution (0.6 mL, 40 mM) in 3.4 mL PO_4_^−3^ buffer. After 10 min, the test compounds' H_2_O_2_ scavenging activity was measured at 230 nm against a blank solution. Under similar conditions, the percentage of inhibition was calculated from the control without the test compound. The IC_50_ values were then calculated using regression analysis.

#### NO Scavenging method

The Griess reaction was used to calculate the amount of NO produced by Na_2_[Fe (CN)_5_NO]^37^. The test chemicals (25, 50, 75, 100, and 125 M) were dissolved in a suitable solvent (CH_3_O_H_) in standard PO_4_^−3^ buffer (0.2 M, pH 7.4) and incubated in the tubes for five hours at 25 °C. The control experiment was carried out similarly to the test experiment but with the same amount of solvent. After 5 h, 0.5 mL of the incubation solution was removed and diluted with 0.5 mL of Griess reagent. At 546 nm, the absorbance of the chromophore formed by nitrite diazotization with sulphanilamide followed by coupling with C_12_H_14_N_2_ was measured. The experiment was repeated three times with Ascorbic acid as the control. The nitric oxide scavenging activity was calculated using the following equation^38^.

$$\mathrm{\% }= \left[\frac{({A}_{Control}-{A}_{Sample})}{{A}_{Control}}\right] \times 100$$

### Computational studies

#### Molecular properties prediction

The molecular characteristics of the synthesized compounds (4a-k) were predicted. Lipinski's rule (L-Rule) of five was used to forecast or estimate whether a chemical molecule predicted or included specific biological activity. L- Rule violations were calculated using topological polar surface area (TPSA), molecular weight, Log P, H-Bond acceptors and donors, number of rotatable bonds of synthesized compounds, and molinspiration online property tool kit^39^. To forecast ADME features such as HIA%, CaCO_2_ permeability, and blood–brain barrier (BBB), the Pre-ADMET web server, http://preadmet.bmdrc.org/, was used.

The ADMET process is vital in determining a medicine's therapeutic efficacy, and drug similarity appears as an important parameter of a compound that optimizes its ADME in the human body. L- Rule of five states that when there are more than 10 H, 5 H- bonds acceptors, and donors, 15 rotatable bonds, a molecular weight larger than 500, and a partition coefficient (Log p) greater than 5, poor absorption or penetration is more likely. Molecules that violate more than one of these characteristics may have issues with bioavailability and drug-likeness^40^. To estimate the drug bioavailability, TPSA is another crucial parameter. The TPSA ≥ 140 Å containing drugs were thought to have low bioavailability^41^.

#### Prediction of bioactivity score

The bioactivity of the synthesized compounds (4a-k) was assessed by using the molinsipartion server to calculate the activity score of drug targets ion channel, kinase, nuclear and GPCR, receptor ligands, protease, and enzyme inhibitors^42^.

#### Molecular docking studies

##### In silico cytotoxic activity

The Schrödinger software suite, which provides a wide range of computational chemistry and molecular modelling methods for studying protein–ligand interactions, was employed in this investigation. These include Glide and Piper molecular docking, FEP + relative binding free energy predictions, Macro Model and Desmond conformational searches, and Prime and Prime X structural refinement^43^.

The docking module in Schrödinger was used to conduct molecular docking studies against the 2IOG protein with compounds 4a-k and the reference Doxorubicin drug. Protein structures were protonated by adding polar hydrogens, and then energy was minimized using the MMFF94x force field to obtain the protein's stable conformer. The inhibitor binding site residues were softened and highlighted using the "Site Finder" module software, and flexible docking was used. The expected grid dimensions for 2IOG were X: 28.27, Y: 27.13, and Z: 28.51. The docking study was conducted with the default settings of placement: triangle matcher, recording 1: London dG, refinement: force field, and the ability to save up to 10 conformations of each compound in a separate mdb format database file^43^.

##### Accession of target protein and doxorubicin

**Figure 6:**
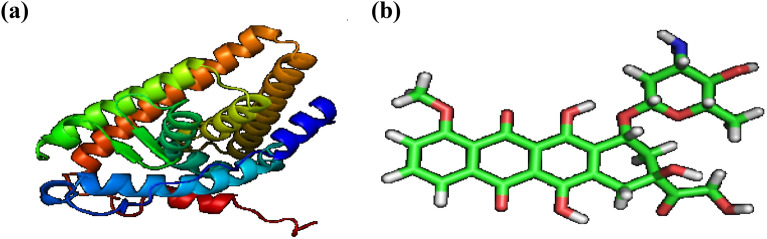
3D structures of (**a**) HERA and (**b**) Doxorubicin.

HERA and Doxorubicin three-dimensional structures were obtained from the RCSB Protein Data Bank and Drug Bank, respectively. The protein's atomic coordinates were separated, and geometry optimization was performed with Argus Lab 4.0.1^44^. Figure 6 (A and B) depict the structures of the target protein and the reference chemicals, 2IOG and Doxorubicin.

##### Ligand preparation

Chem Bio Draw^45^ was used to generate compound molecular structures, which converted all ligands into Pdbqt file format and calculated atomic coordinates using the Schrödinger module.

##### Analysis of target active binding sites

The target protein's active sites are represented by the ligand's coordinates in the original target protein grids. These target protein binding sites were examined using the virtual instruments 3D Ligand Site and Drug Discovery Studio version 3.0^46,47^.

##### Structural analysis and visualization

Protein and ligand interactions have been studied and visualized using the Pymol Viewer tool (www.pymol.org)^48^.

### In Silico Antioxidant activity: Accession of the target protein and reference drugs

**Figure 7:**
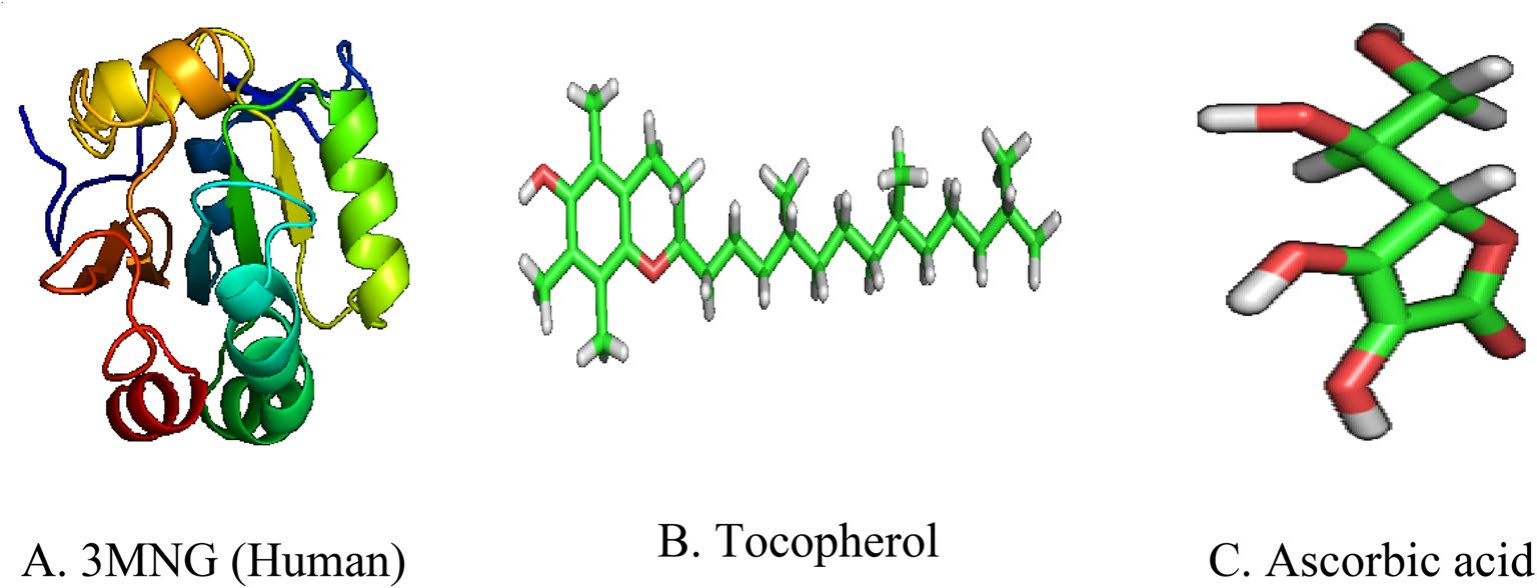
Structures of the target protein and reference compounds.

Peroxiredoxins (PDB: 3MNG) and reference medications such as tocopherol (Pub Chem ID 14,986) and ascorbic acid (Pub Chem ID 54,670,067) were downloaded from the RCSB Protein Data Bank and Pub Chem. After separating the atomic coordinates of the protein, Argus Lab 4.0.1 was used to optimize its geometry^44^. Figure 7 (A, B, and C) depicts the enzyme 3MNG as well as reference medications such as Tocopherol and Ascorbic acid.

### Molecular docking analysis

The 3MNG protein was molecular docked utilizing compounds 4a-k and the reference medication, Ascorbic acid and tocopherol, as well as the Schrödinger docking module. The protein structures were protonated by adding polar hydrogens, and then energy was reduced using the MMFF94x force field to obtain the protein's stable conformer. Using the "Site Finder" module software, the inhibitor binding site residues were softened and highlighted, and flexible docking was used. Aromatase's grid dimensions were predicted to be X: 28.27, Y: 27.13, and Z: 28.51. The default parameters for the docking research were placement: triangle matcher, recording 1: London dG, refinement: force field, and a maximum of 10 conformations of each compound allowed to be saved in a separate database file in mdb format^43^.

## Conclusion

Biologically potent chromone derivatives of *N*-(4-oxo-2-(trifluoromethyl)-4*H*-chromen-7-yl) benzamides (**4a-k**) were synthesized at good yields with shorter reaction times. The titled compounds were assessed for cytotoxic activity against cell lines, such as the human lung adenocarcinoma cell line (A-549) and breast cancer cell line (MCF-7). Among the synthesized compounds, compound **4h** having a *p*-fluorophenyl substitution at the chromenbenzamide side chain showed good cytotoxic activity, good DPPH radical scavenging, TAC, nitric oxide scavenging, hydrogen peroxide scavenging activities. The SAR studies performed on the new chromone derivatives revealed that the 4*H*-chromo benzamide core unit itself has a good bioactivity and, in addition to this present study designed and introduced another bio-potent trifluoromethyl group on it as a best anchoring group in pharmacology, which might be useful as an anticancer candidate with good oral bioavailability. Comparative analysis with previous or contemporary studies reinforces the novelty and significance of the findings. The results align well with emerging trends in drug design, emphasizing the importance of rational molecular modifications to enhance therapeutic efficacy. Furthermore, the molecular docking studies conducted on the synthesized compounds revealed promising binding interactions with specific targets (HERA and 3MNG), thereby providing valuable insights into the potential mechanisms of action. S*ilico* studies titled compounds showed good binding interactions with the targets (HERA and 3MNG), suggesting a good correlation between molecular docking and in vitro studies. The integration of in vitro cytotoxicity assays with computational modelling enhances the credibility and translational relevance of the study findings. The observed correlation between molecular docking predictions and experimental results underscores the reliability of the proposed molecular design strategies. The synthesized chromone derivatives show great potential as cytotoxic drugs, poised for further development into effective anticancer agents. Their favourable pharmacological profile, supported by strong in vitro and computational evidence, makes them promising candidates for clinical trials. This research expands the range of bioactive chromone derivatives and emphasizes the role of rational drug design in advancing novel anticancer therapies, contributing significantly to cancer research.

### Supplementary Information

Supplementary Figures.

## Data Availability

No data other than those reported in this manuscript are available.
