# Supplementary material for: Synthesis, molecular docking studies and biological evaluation of N-(4-oxo-2-(trifluoromethyl)-4H-chromen-7-yl) benzamides as potential antioxidant, and anticancer agents
Source: Sci Rep. 2024 Apr 29;14:9866. doi: 10.1038/s41598-024-59166-5 (PMC11058781; doi:10.1038/s41598-024-59166-5)
Supplement: Supplementary file 1 — Supplementary Figures. [file 41598_2024_59166_MOESM1_ESM.pdf]

Supplementary material

Synthesis, molecular docking studies and biological evaluation of *N*-(4-oxo-2-(trifluoromethyl)-4*H*-chromen-7-yl) benzamides as potential antioxidant, and anticancer agents

J. Sumalatha[a,b], A. Sreedevi[a*], C. Radha Rani[c], G. Chandra Sekhar Reddy[c], Janardhan Reddy Koduru [d]*, Rama Rao Karri [e]*

[a] Department of Pharmaceutical Chemistry, Sri Padmavati Mahila Visva Vidyalayam, Tirupati, 517 502, India.

[b] Department of Pharmaceutical Chemistry, P.R. Reddy Memorial College of Pharmacy, Kadapa-516 003, India

[c] Department of Chemistry, Sri Venkateswara College of Engineering, Tirupati-517 507, India

[d] Department of Environmental Engineering, Kwangwoon University, Seoul 01897, Republic of Korea

[e] Petroleum and Chemical Engineering, Faculty of Engineering, Universiti Teknologi Brunei, Bandar Seri Begawan, BE1410, Brunei Darussalam

*Corresponding author: e-mail: sreedevi@spmvv.ac.in (AS); kramarao.iitd@gmail.com (RRK); reddyjchem@gmail.com (JRK)

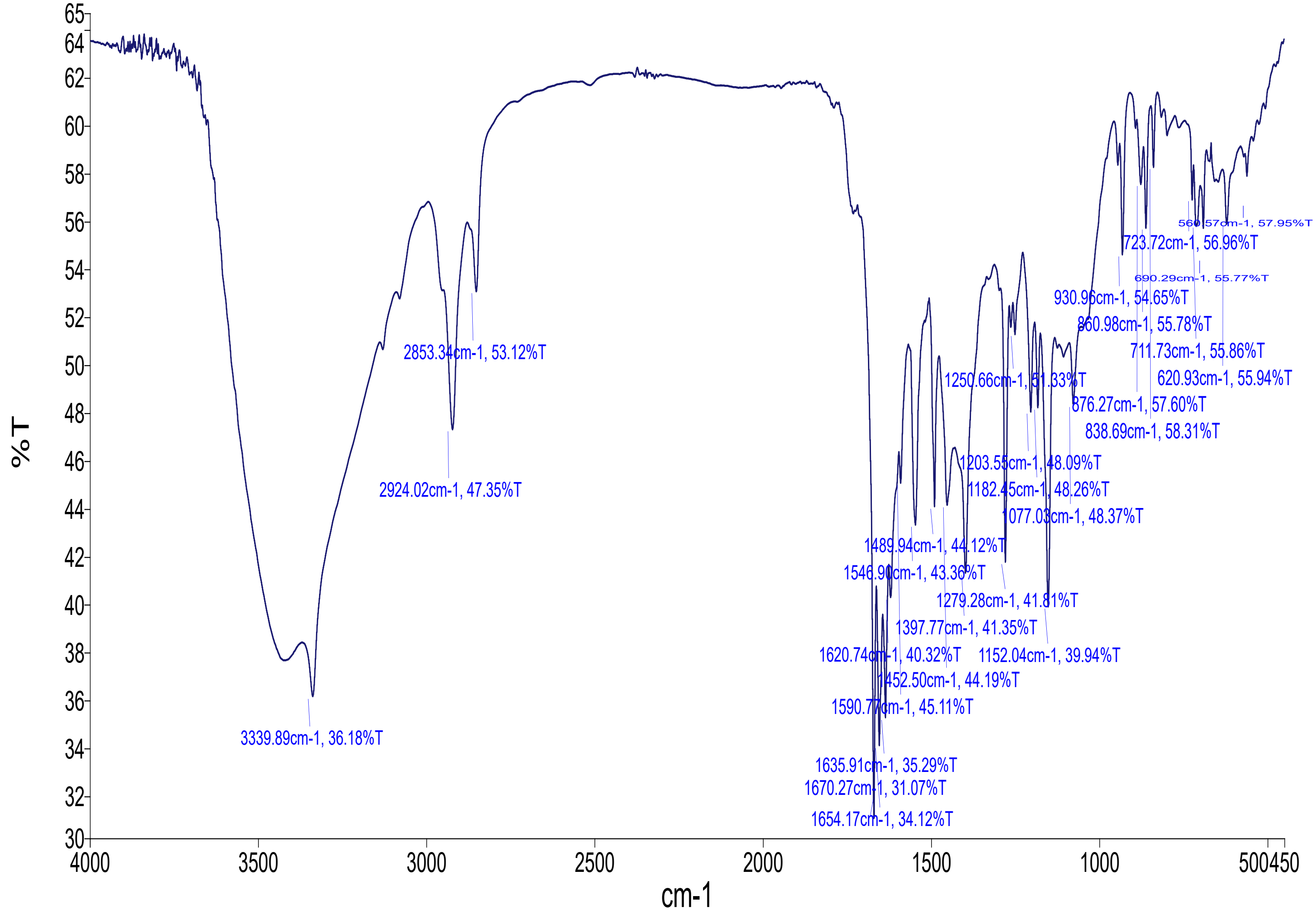

Figure 1. IR spectrum of *N-(4-oxo-2-(trifluoromethyl)-4H-chromen-7-yl)benzamide* (4c).

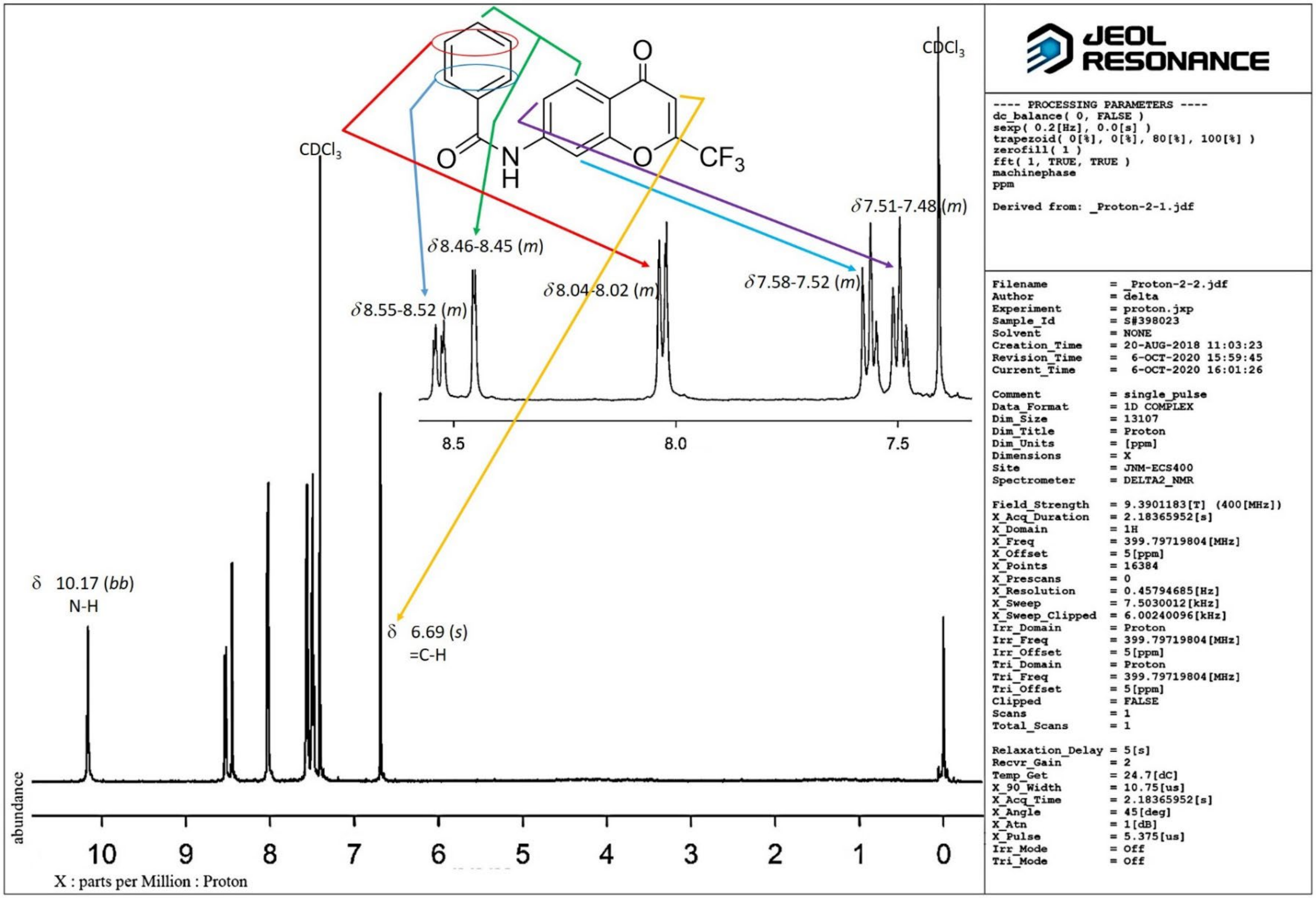

Figure 2. **^{1}H-NMR Spectrum of** ***N-(4-oxo-2-(trifluoromethyl)-4H-chromen-7-yl)benzamide*** **(4c)**

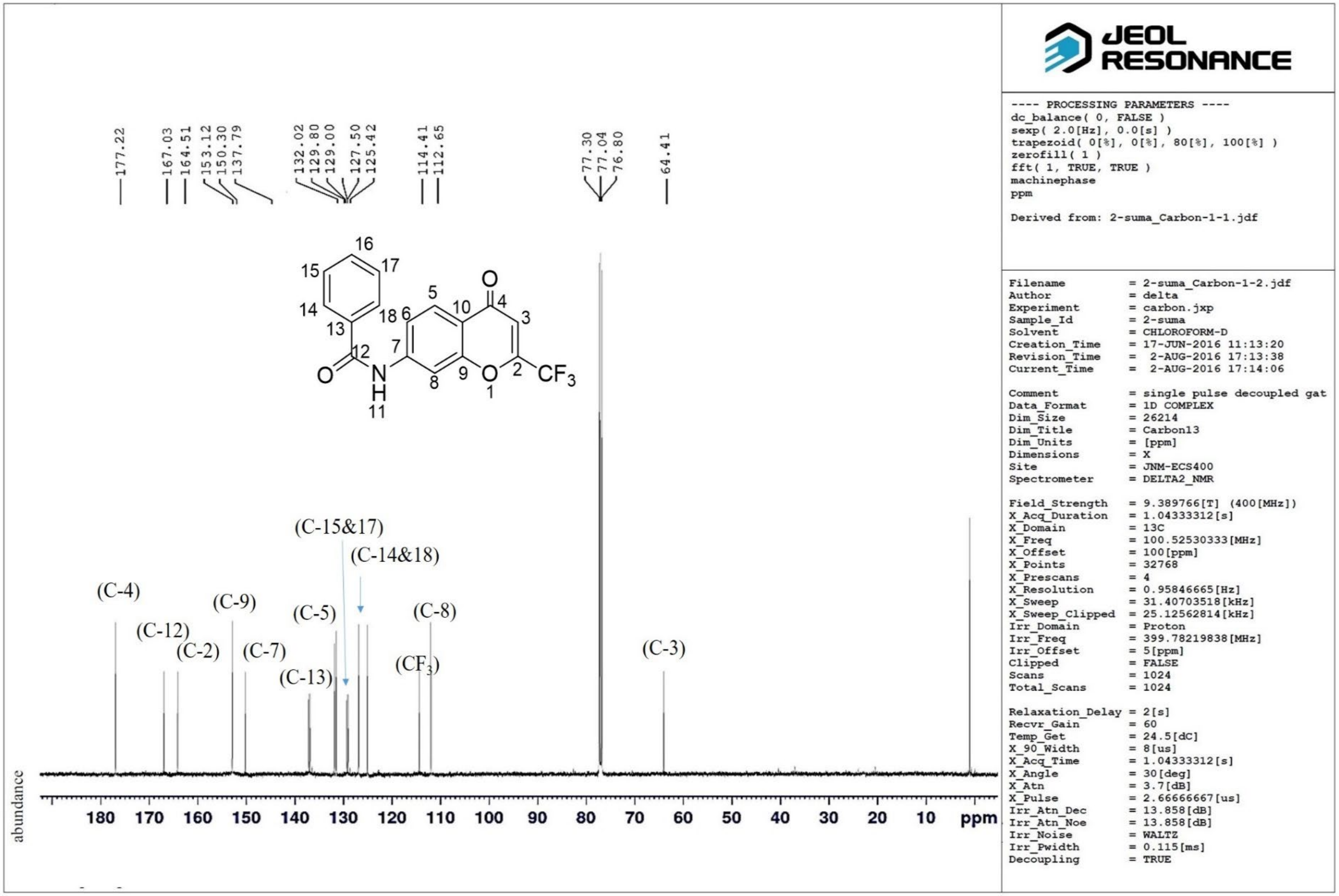

Figure 3. ^{13}**C-NMR Spectrum of** ***N-(4-oxo-2-(trifluoromethyl)-4H-chromen-7-yl)benzamide*** **(4c)**

LCMS-2010A DATA REPORT
SHIMADZU

User	: Admin
Sample	: S2
Inj. Volume	: 5.000
Data Name	: C:\LCMSsolution\User\Data\S2-APCI-POS1.qld
Method Name	: C:\LCMSsolution\User\Method\esi.qlm

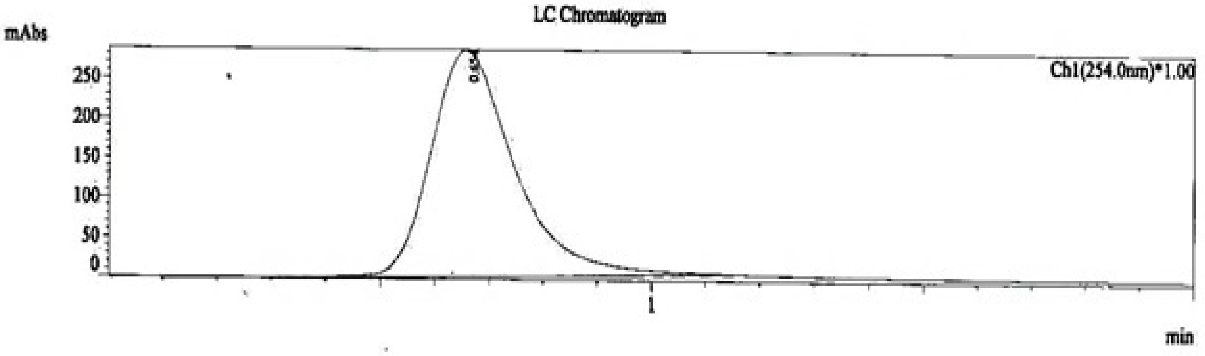

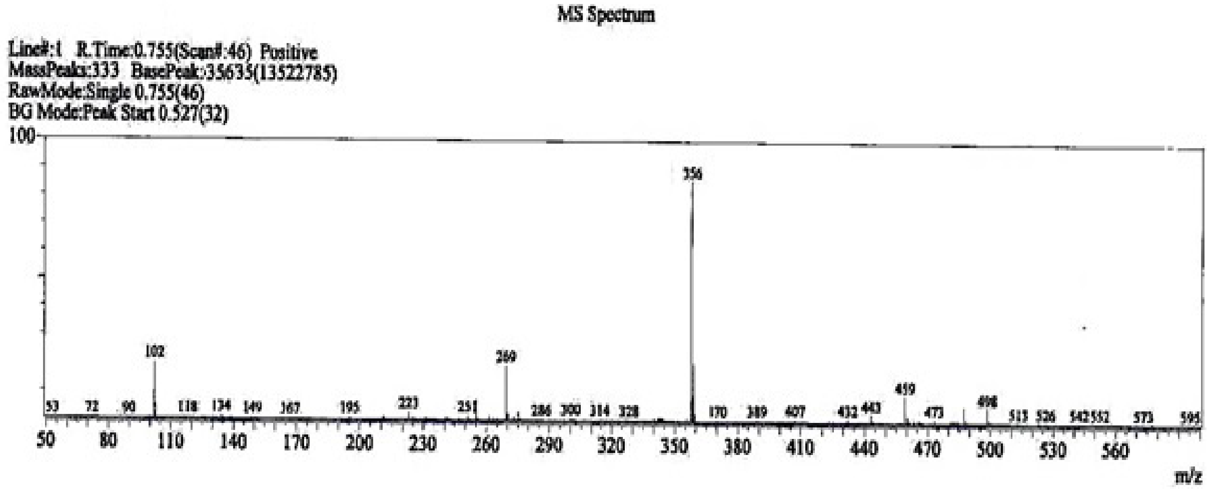

Figure 4. Mass spectrum of ***N-(4-oxo-2-(trifluoromethyl)-4H-chromen-7-yl)benzamide*** (**4c**)

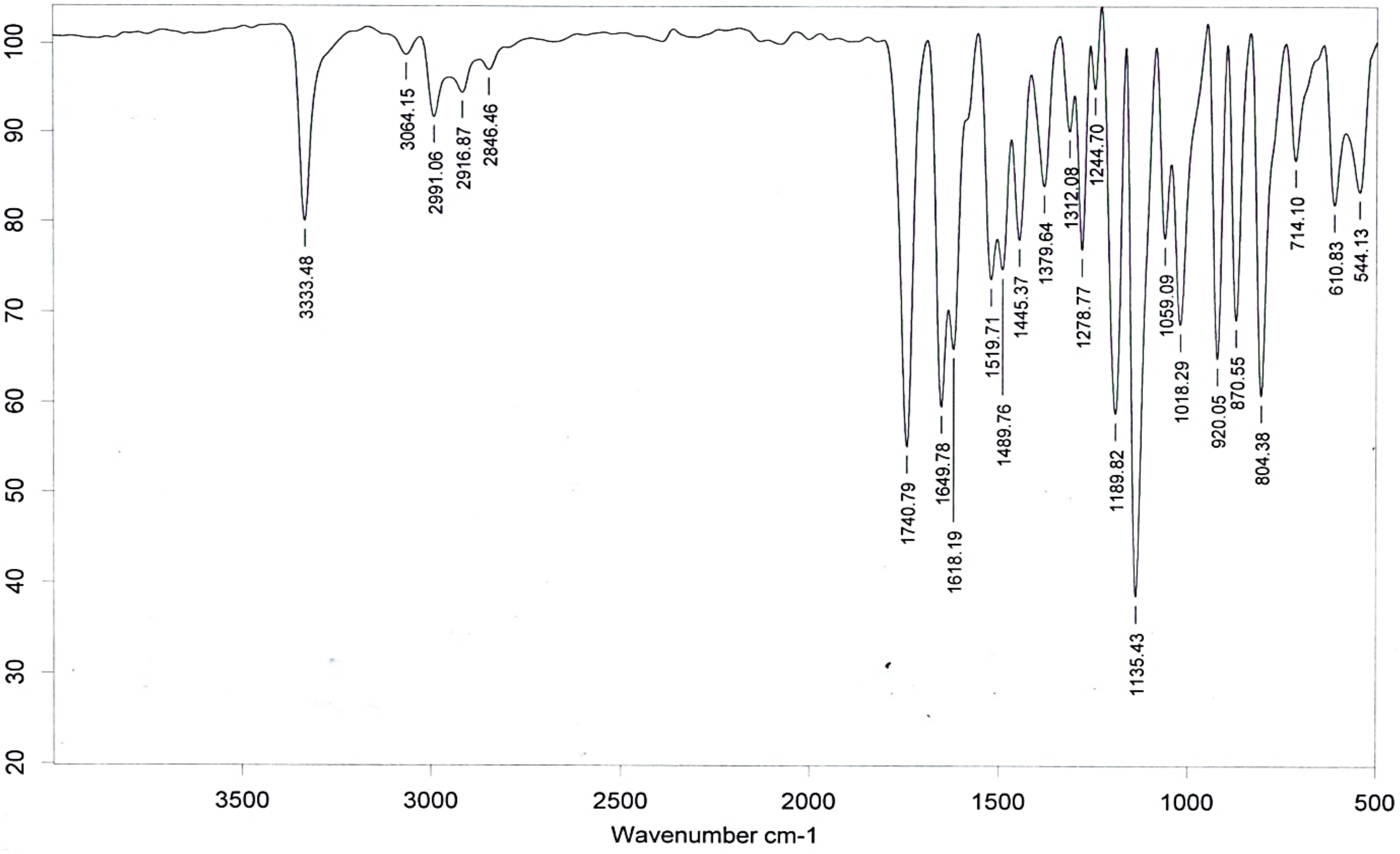

Figure 5. IR spectrum of ***N-(4-oxo-2-(trifluoromethyl)-4H-chromen-7-yl)acetamide*** **(4d).**

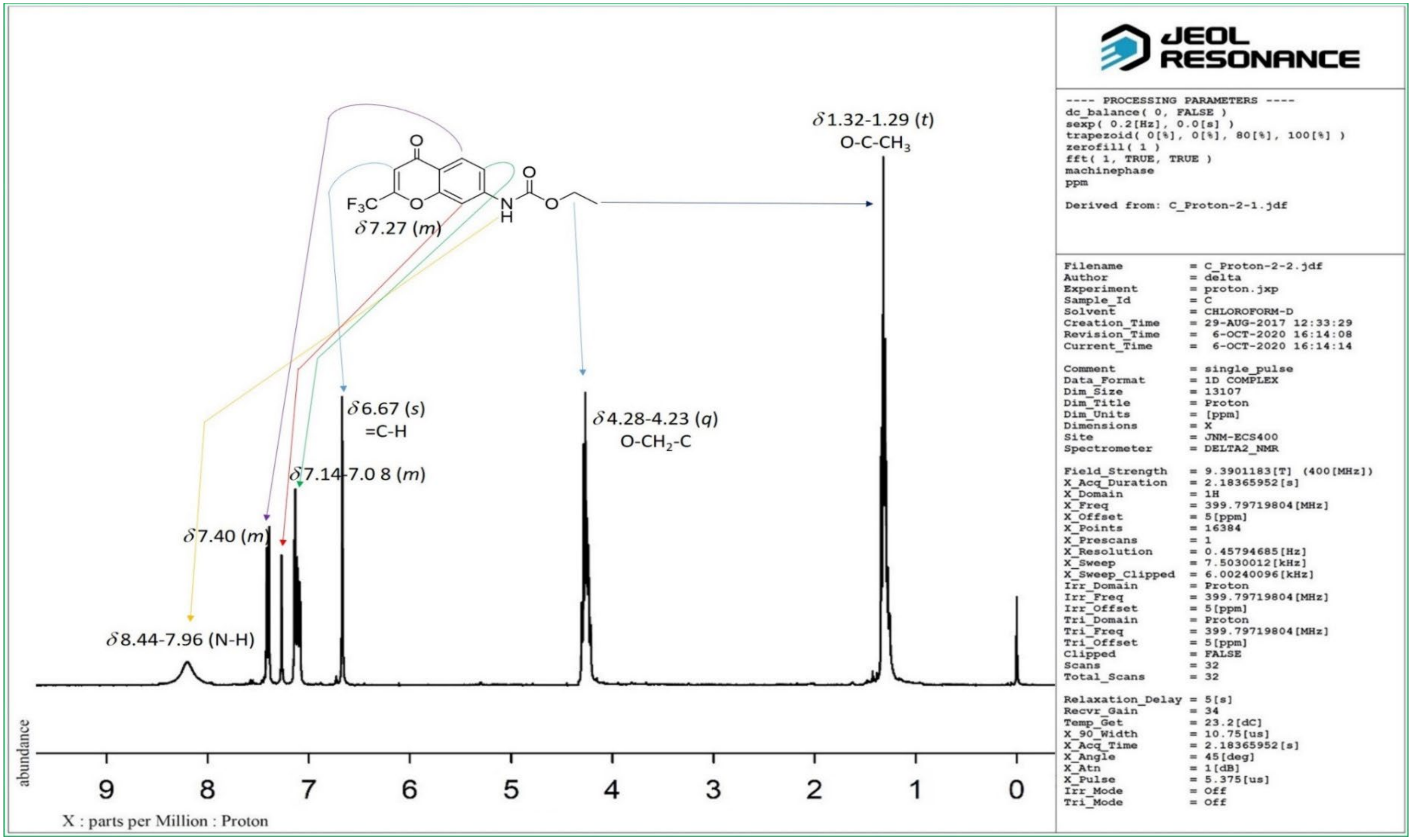

Figure 6. ^{1}H-NMR Spectrum of ***N-(4-oxo-2-(trifluoromethyl)-4H-chromen-7-yl)acetamide*** **(4d).**

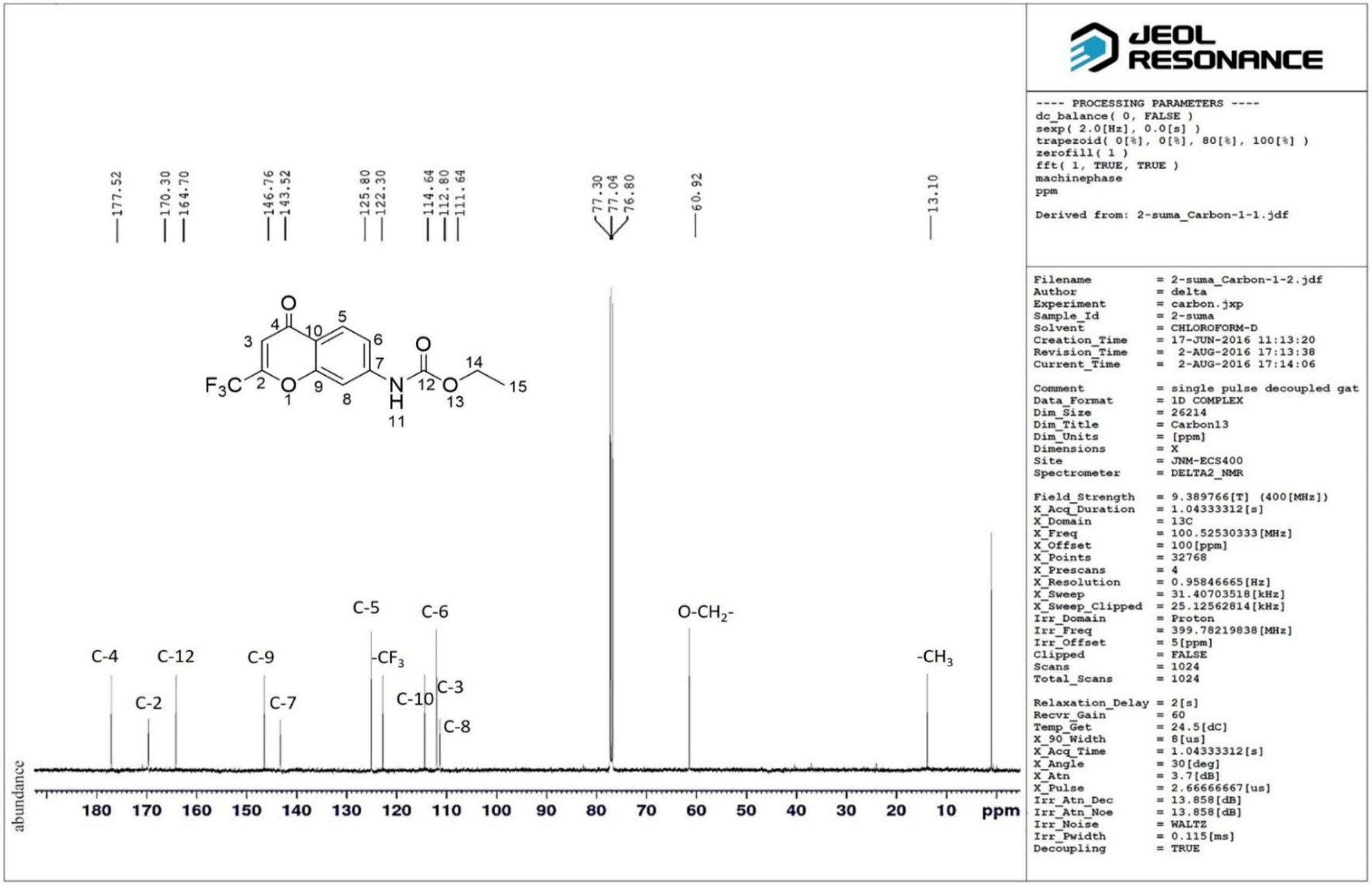

Figure 7. ^{13}C-NMR Spectrum of ***N-(4-oxo-2-(trifluoromethyl)-4H-chromen-7-yl)acetamide*** **(4d).**

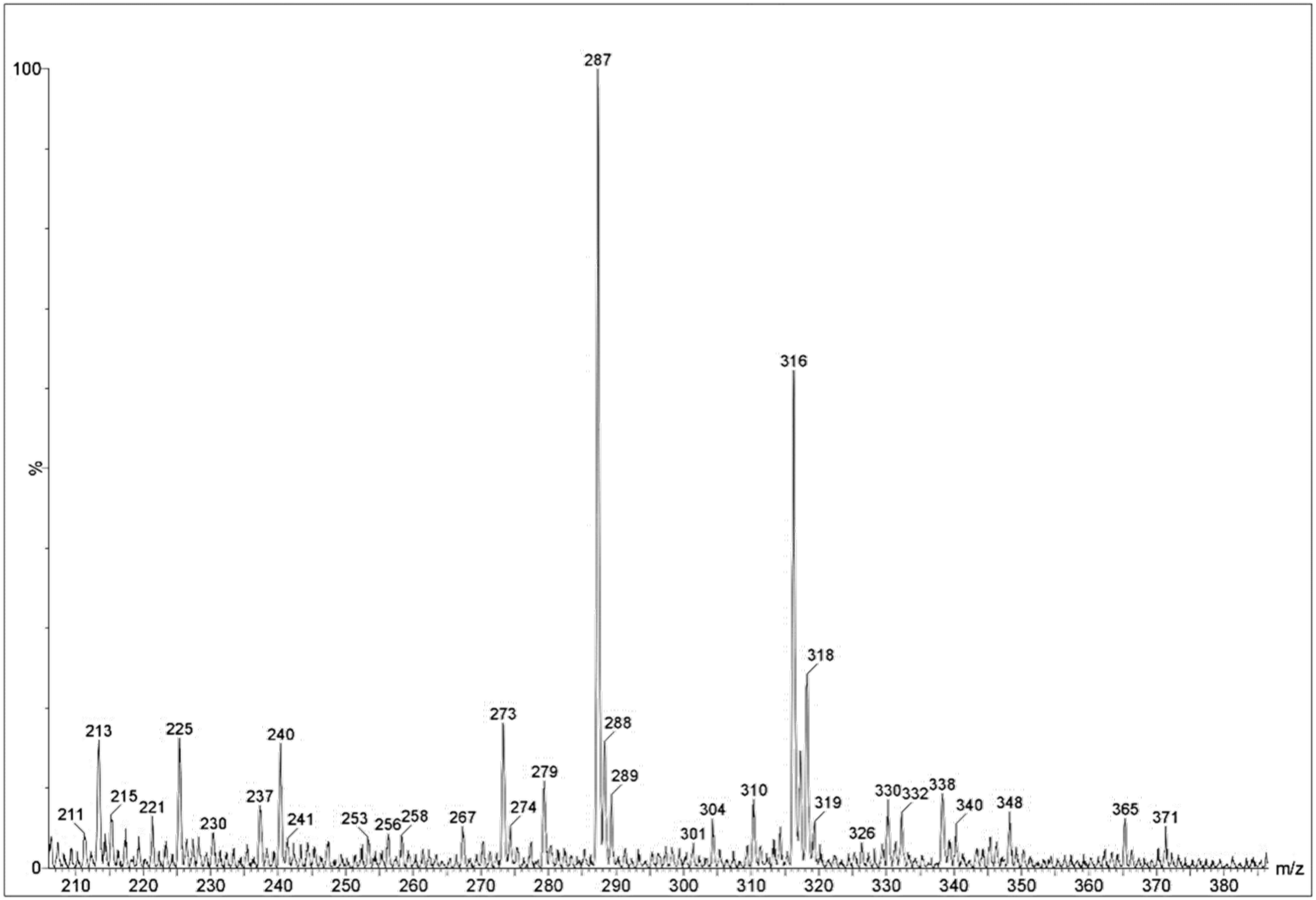

Figure 8. Mass spectrum of the compound *N-(4-oxo-2-(trifluoromethyl)-4H-chromen-7-yl)acetamide* **(4d)**.

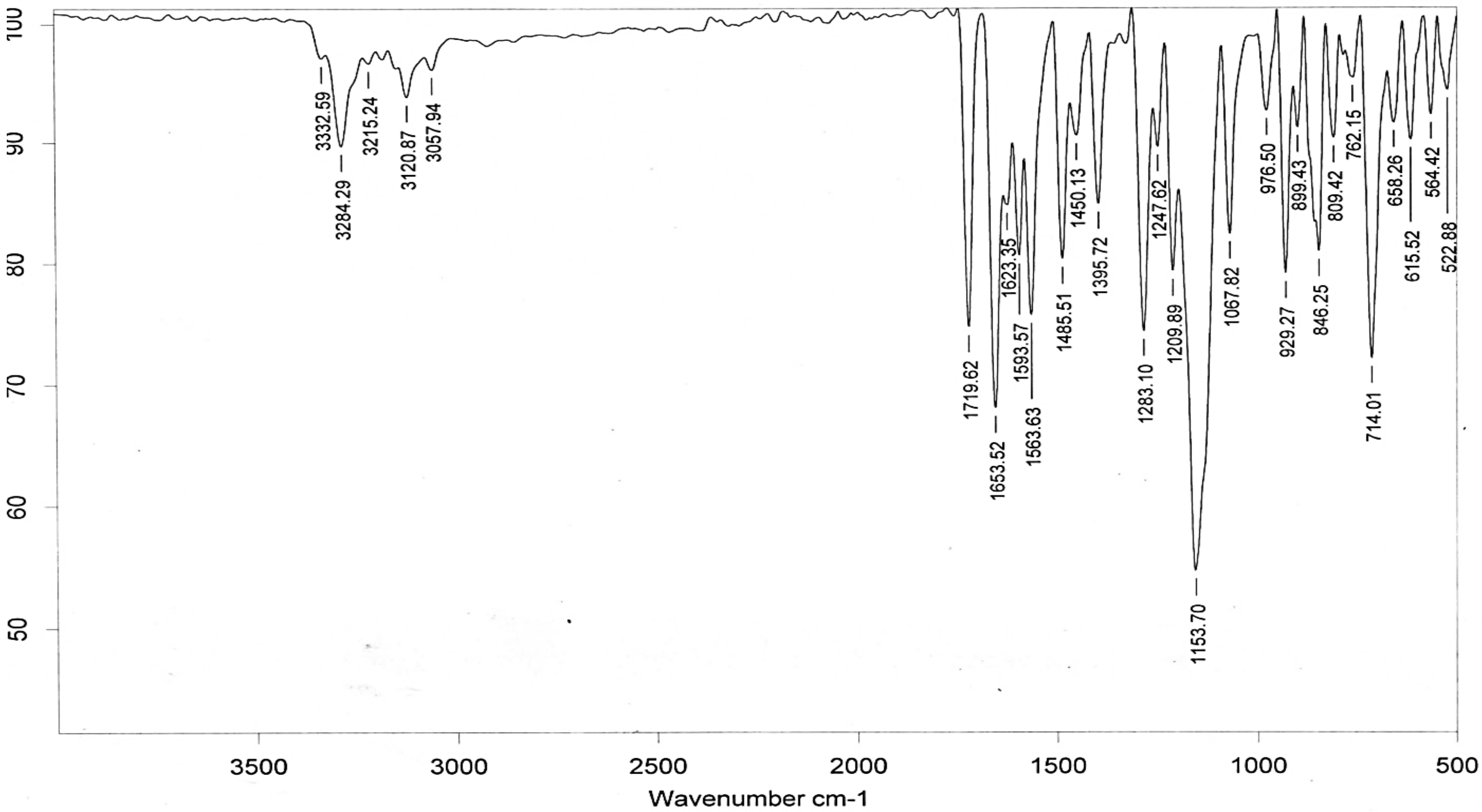

:\OC USERS\scn lab\BRK-1-KL-V - NEAT

Figure 9. IR spectrum of ***2,2,2-trifluoro-N-(4-oxo-2-(trifluoromethyl)-4H-chromen-7-yl)acetamide*****(4e).**

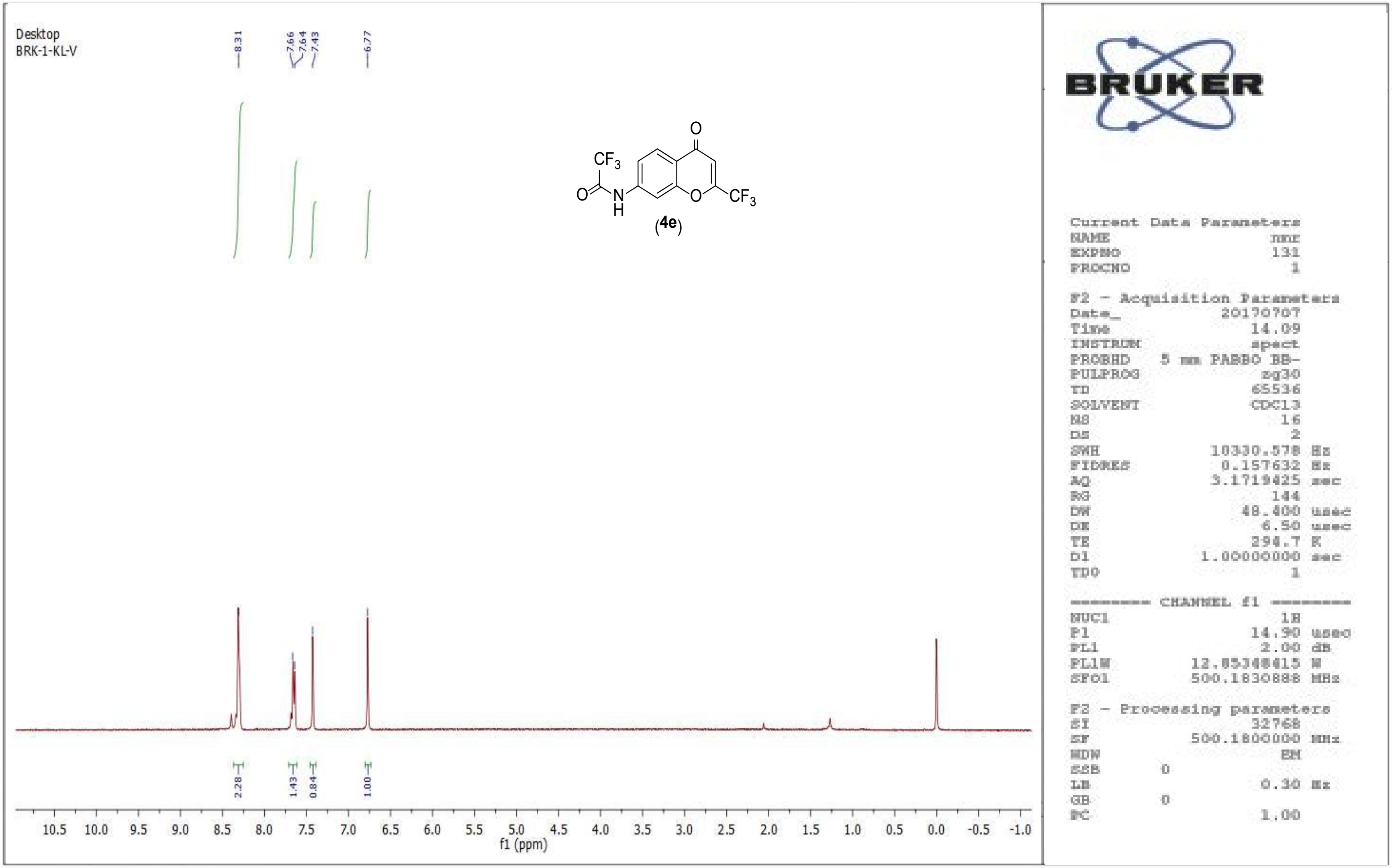

Figure 10. ^{1}H-NMR Spectrum of ***2,2,2-trifluoro-N-(4-oxo-2-(trifluoromethyl)-4H-chromen-7-yl)acetamide*** **(4e).**

Physical and Spectral Characterization of Synthesized Compounds (4a-k).

1) 4-methyl-*N*-(4-oxo-2-(trifluoromethyl)-4*H*-chromen-7-yl)-*N*-tosylbenzenesulfonamide (4a).

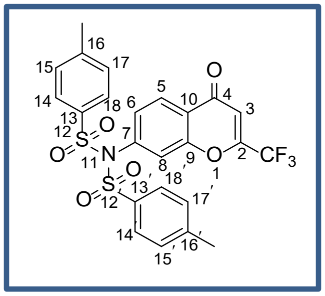

IR (KBr) ν_{max}: 3078.8, 3030.3 (C=C$_{Stretch}$), 2926.2 & 2855.7 (C-H$_{Stretch}$), 1675.9 (C=O), 1602.4 (C-), 1476.1 & 1449.0 (C-H$_{Bend}$), 1380.1 (S=O$_{Stretch}$) cm^{-1}; ^{1}H NMR (CDCl$_3$) δ: 7.87 (4H, *d*, $^{3}J_{H\text{-}H}$ = 8.0 Hz, Ar-H$_{(14,14',18\&18')}$), 7.78 (4H, *d*, $^{3}J_{H\text{-}H}$ = 8.0 Hz, Ar-H$_{(15,15',17\&17')}$), 7.58-7.56 (1H, *d*, $^{3}J_{H\text{-}H}$ = 10.0 Hz, Ar-H$_{(5)}$), 7.54 (1H, *s*, =CH$_{(3)}$), 6.85-6.76 (2H, *m*, Ar-H$_{(6\&8)}$); ^{13}C NMR (CDCl$_3$) δ: 177.1 (C-4), 160.4 (C-2), 149.2 (C-9), 145.3 (C-7), 124.8 (C-16&16′), 122.5 (C-13&13′), 119.4 (C-5), 109.4 (C-15,15′,17&17′), 103.6 (C-14,14′,18&18′), 98.9 (-CF$_3$), 95.1 (C-8), 61.6 (C-3), 14.1 (-CH$_3$); Mass [M+Na]$^{+\bullet}$ = 560.

2) 4-Nitro-*N*-(4-oxo-2-(trifluoromethyl)-4*H*-chromen-7-yl)benzamide (4b)

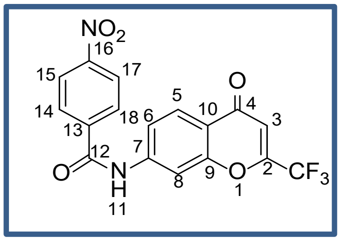

IR (KBr) ν_{max}: 3308.3 (N-H$_{Stretch}$), 3097 (C=C$_{Stretch}$), 2920.2 & 2850.8 (C-H$_{Stretch}$), 1651.9 (C=O), 1524.6 & 1486.9 (C-H$_{Bend}$) cm^{-1}; ^{1}H NMR (CDCl$_3$) δ: 8.56-8.52 (1H, *bb*, N-H), 8.29-8.27 (2H, *m*, Ar-H$_{(15\&17)}$), 8.11-8.03 (2H, *m*, Ar-H$_{(14\&18)}$), 7.58-7.56 (1H, *d*, $^{3}J_{H\text{-}H}$ = 10.1 Hz, Ar-H$_{(5)}$), 7.51 (1H, *s*, =CH$_{(3)}$), 6.95-6.83 (2H, *m*, Ar-H$_{(6\&8)}$); ^{13}C NMR (CDCl$_3$) δ: 177.3 (C-4), 160.1 (C-2), 149.5 (C-9), 146.1 (C-16), 145.0 (C-7), 124.9 (C-13), 122.9 (C-5), 119.4 (C-14&18), 115.4 (C-15&17), 103.6 (-CF$_3$), 95.6 (C-8), 61.5 (C-3); Mass [M+1]$^{+\bullet}$ = 379.

3) *N*-(4-oxo-2-(trifluoromethyl)-4*H*-chromen-7-yl)benzamide (4c).

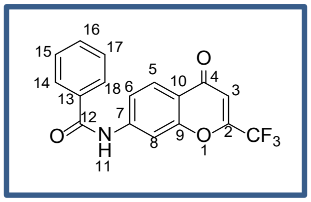

IR (KBr) v_{max}: 3339.8 (N-$H_{Stretch}$), 2924.0 & 2853.3 (C=$C_{Stretch}$), 1670.2 (C=O) cm^{-1}; ^{1}H NMR ($CDCl_3$) δ: 10.17 (1H, *bb*, N-H), 8.55-8.52 (2H, *m*, Ar-$H_{(14\&18)}$), 8.46-8.45 (2H, *m*, Ar-$H_{(5\&16)}$), 8.04-8.02 (2H, *m*, Ar-$H_{(15\&17)}$), 7.58-7.52 (1H, *m*, Ar-$H_{(8)}$), 7.51-7.48 (1H, *m*, Ar-$H_{(7)}$), 6.69 (1H, *s*, =$CH_{(3)}$); ^{13}C NMR ($CDCl_3$) δ: 177.2 (C-4), 167.0 (C-12), 164.5 (C-2), 153.1 (C-9), 150.3 (C-7), 137.7 (C-13), 132.0 (C-5), 129.8 & 129.0 (C-15&17), 127.5 & 125.4 (C-14&18), 114.4 (-CF_3), 112.6 (C-8), 64.4 (C-3); Mass $[M+Na]^{+\bullet}$ = 356, $[M]^{+\bullet}$ = 333; Anal. Calcd. for $C_{17}H_{10}F_3NO_3$: C, 61.27; H, 3.02; N, 4.20; found: C, 61.25; H, 3.01; N, 4.21.

4) *N*-(4-oxo-2-(trifluoromethyl)-4*H*-chromen-7-yl)acetamide (4d).

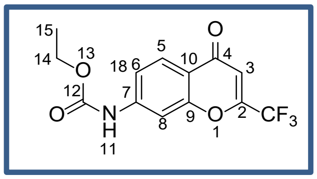

IR (KBr) v_{max}: 3333.4 (N-H), 1740.7 (C=O), 1649.7 (C=O), 1135 (C-F) cm^{-1}; ^{1}H NMR ($CDCl_3$) δ: 8.44-7.96 (1H, *bb*, N-H), 7.40 (1H, *m*, Ar-$H_{(5)}$), 7.27 (1H, *m*, Ar-$H_{(8)}$), 7.14-7.08 (1H, *m*, Ar-$H_{(6)}$), 6.67 (1H, *s*, =$CH_{(3)}$), 4.28-4.23 (2H, *q*, $^3J_{H\text{-}H}$ = 8.0 Hz, (O-$CH_{2(14)}$), 1.33-1.29 (3H, *t*, $^3J_{H\text{-}H}$ = 8.0 Hz, (-$CH_{3(15)}$); ^{13}C NMR ($CDCl_3$) δ: 177.5 (C-4), 170.3 (C-2), 164.7 (C-12), 146.7 (C-9), 143.5 (C-7), 125.8 (C-5), 122.3 (-CF_3), 114.6 (C-10), 112.8 (C-6&C-3), 111.6 (C-8), 60.9 (O-CH_2-), 13.1 (-CH_3.

5) 2,2,2-trifluoro-*N*-(4-oxo-2-(trifluoromethyl)-4*H*-chromen-7-yl)acetamide (4e).

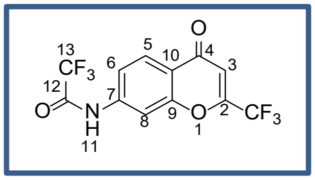

IR (KBr) v_{max}: 3284.2 (N-H), 3215.2 (C=C), 1719.6 (C=O), 1653.5 (C=O), 1153 (C-F) cm^{-1}; ^{1}H NMR ($CDCl_3$) δ: 8.31 (1H, *bb*, N-H),

7.65 (1H, *m*, Ar-$H_{(5)}$), 7.43 (2H, *m*, Ar-$H_{(6\&8)}$), 6.77 (1H, *s*, =$CH_{(3)}$); ^{13}C NMR ($CDCl_3$) δ: 177.1, 171.4, 162.5, 152.5, 146.2, 126.8, 126.2, 122.4, 118.4, 112.4, 111.6, 61.6 (C-3.

6) *N*-(4-oxo-2-(trifluoromethyl)-4*H*-chromen-7-yl)acetamide (4f).

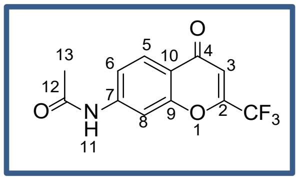

IR (KBr) ν_{max}: 3336, 1648.6 (C=O), 1142 cm^{-1}; ^1H NMR ($CDCl_3$) δ: 8.02 (1H, *bb*, N-H), 7.99 (1H, *m*, Ar-$H_{(5)}$), 7.56 (2H, *m*, Ar-$H_{(6\&8)}$), 6.78 (1H, *s*, =$CH_{(3)}$), 2.05 (3H, *s*, -CH_3); ^{13}C NMR ($CDCl_3$) δ: 177.12, 170.43, 149.25, 145.30, 124.82, 122.48, 119.42, 109.45, 103.65, 61.5 (C-3), 45.67, 14.12.

7) *N*-(4-oxo-2-(trifluoromethyl)-4*H*-chromen-7-yl)methanesulfonamide (4g).

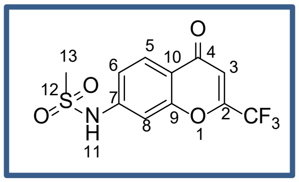

IR (KBr) ν_{max}: 3078, 2926, 1675.7 (C=O), 1351.3 ($S{=}O_{Stretch}$) cm^{-1}; ^1H NMR ($CDCl_3$) δ: 8.22 (1H, *bb*, N-H), 7.76-7.68 (3H, *m*, Ar-$H_{(5,6\&8)}$), 6.80 (1H, *s*, =$CH_{(3)}$), 3.46 (3H, *s*, -CH_3); ^{13}C NMR ($CDCl_3$) δ: 177.12, 170.43, 149.25, 145.30, 124.82, 122.48, 119.42, 109.45, 103.65, 61.5 (C-3), 45.67, 14.12.

8) 4-fluoro-*N*-(4-oxo-2-(trifluoromethyl)-4*H*-chromen-7-yl)benzamide (4h).

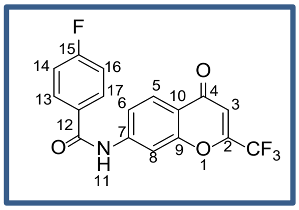

IR (KBr) ν_{max}: 3078, 2360, 1652.5 (C=O), 1151 cm^{-1}; ^1H NMR ($CDCl_3$) δ: 8.21 (1H, *bb*, N-H), 8.10 (2H, *m*, Ar-$H_{(13\&17)}$), 7.75 (1H, *m*, Ar-$H_{(5)}$), 7.38 (2H, *m*, Ar-$H_{(14\&16)}$); 7.27 (2H, *m*, Ar-$H_{(6\&8)}$), 6.50 (1H, *s*, =$CH_{(3)}$); ^{13}C NMR ($CDCl_3$) δ: 177.12, 170.43, 149.25, 145.30, 124.82, 122.48, 119.42, 109.45, 103.65, 61.56, 45.67, 14.12.

9) 2-chloro-*N*-(4-oxo-2-(trifluoromethyl)-4*H*-chromen-7-yl)acetamide (4i).

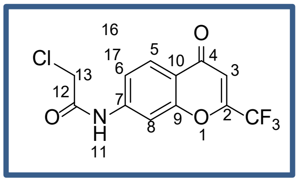

IR (KBr) ν_{max}: 3078, 2926, 1675.7 (C=O) cm^{-1}; ^{1}H NMR ($CDCl_3$) δ: 8.58 (1H, *bb*, N-H), 8.36-8.33 (1H, *m*, Ar-$H_{(5)}$), 8.13 (1H, *m*, Ar-$H_{(6)}$), 7.62 (1H, *m*, Ar-$H_{(8)}$), 6.75 (1H, *s*, =$CH_{(3)}$), 4.26 (2H, *s*, -$CH_{2(13)}$-); ^{13}C NMR ($CDCl_3$) δ: 177.12, 170.43, 149.25, 145.30, 124.82, 122.48, 119.42, 109.45, 103.65, 61.56, 45.67, 14.12.

10) *N*-(4-Oxo-2-(trifluoromethyl)-4*H*-chromen-7-yl)propionamide (4j).

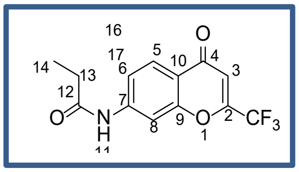

IR (KBr) νmax: 3385 (N-H), 3098 & 2920, 1651.8 (C=O) cm-1; 1H NMR (CDCl3) δ: 8.02 (1H, bb, N-H), 7.99 (1H, m, Ar-H(5)), 7.56 (2H, m, Ar-H(6&8)), 6.78 (1H, s, =CH(3)), 2.05-2.02 (2H, q, 3JH-H = 7.8 Hz, -CH2(13)-), 1.14-1.11 (3H, t, 3JH-H = 7.8 Hz, -CH3(14)); 13C NMR (CDCl3) δ: 177.12, 170.43, 149.25, 145.30, 124.82, 122.48, 119.42, 109.45, 103.65, 61.56, 45.67, 14.12.

11) *N*-(4-oxo-2-(trifluoromethyl)-4*H*-chromen-7-yl)butyramide (4k)

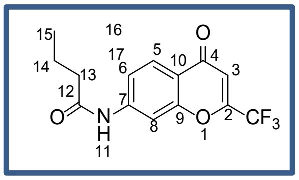

IR (KBr) ν_{max}: 3078, 2926, 1675.3 (C=O) cm^{-1}; ^{1}H NMR ($CDCl_3$) δ: 8.51-8.48 (1H, *bb*, N-H), 8.01 (1H, *m*, Ar-$H_{(5)}$), 7.82 (2H, *m*, Ar-$H_{(6\&8)}$), 7.58 (1H, *s*, =$CH_{(3)}$), 2.47-2.44 (2H, *m*, $^{3}J_{H-H}$ = 7.4 Hz, -$CH_{2(13)}$-), 1.77-1.74 (2H, *m*, -$CH_{2(14)}$-), 1.44-1.00 (3H, *t*, $^{3}J_{H-H}$ = 7.8 Hz, -$CH_{3(15)}$); ^{13}C NMR ($CDCl_3$) δ: 177.12, 170.43, 149.25, 145.30, 124.82, 122.48, 119.42, 109.45, 103.65, 61.56, 45.67, 14.12.

Table S1. Synthesis of 4-oxo-2-(trifluoromethyl)-4*H*-chromen-7-yl)benzamide derivatives (**4a-k**).

Compound	Product	Yield (%)	mp (ºC)
**4a**	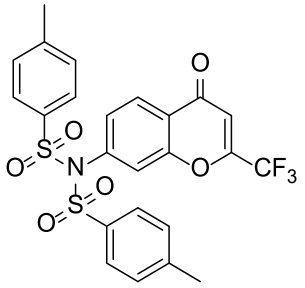	95	192-194
**4b**	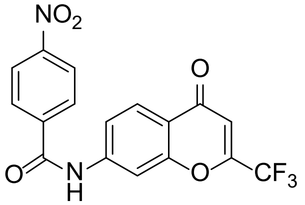	96	213-215
**4c**	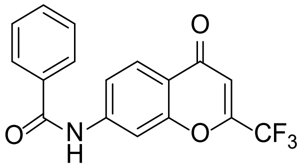	91	186-188
**4d**	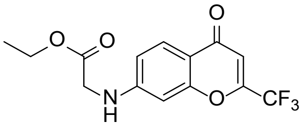	88	163-165
**4e**	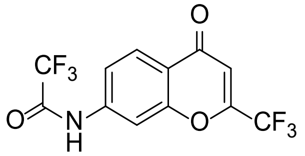	89	169-171
**4f**	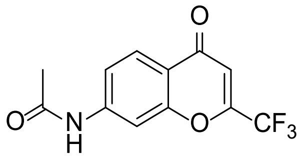	93	155-157

**4g**	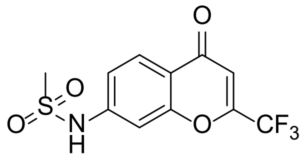	90	201-203
**4h**	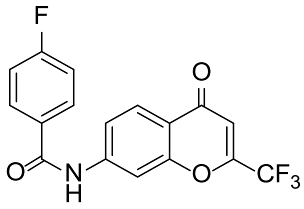	91	197-199
**4i**	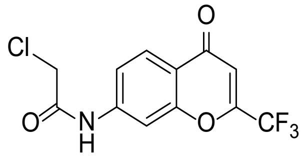	90	165-167
**4j**	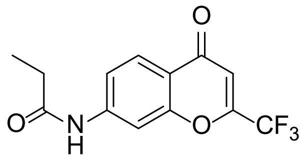	87	168-170
**4k**	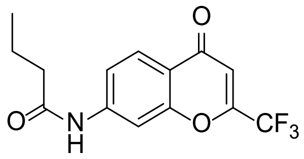	93	158-160

2.3. Computational studies

2.3.1 Molecular Properties Prediction

Table S2 contains a tabulation of the title compound's computed molecular characteristics. The majority of synthesized derivatives have zero or one violation rate. The obtained results proved that every synthetic derivative exhibited good drug-like properties and complied with Lipinski's criteria. The compounds have good oral bioavailability, as shown by the TPSA of less than 140.

BBB (blood-brain barrier penetration) and the amount of intestinal absorption in humans (HIA%) were projected for each derivative, in addition to the other molecular predictions. All the compounds were found to be well absorbed and to have high HIA% values between 94.54% and 98.58%. Furthermore, it was discovered that they had a moderate BBB-to-CNS penetration rate (0.02-3.63). Therefore, theoretically, all of these parameters showed that the compounds had adequate oral absorption, bioavailability, and reasonable permeability through the blood-brain barrier.

2.3.2. Bioactivity score prediction

Table S2 summarises the computed bioactivity scores of the compounds that were screened for GPCR ligand, kinase, protease, and enzyme inhibitors. The synthesized compounds exhibited good to moderate behaviour towards inhibitors, as indicated by their bioactive score. The titled compounds exhibited high bioactivity scores of -0.12 to -0.38 towards enzyme inhibitors. It was observed from the bioactivity scores of compounds that were found within the range of -0.12 to -0.73.

Table S2. Prediction of molecular properties, bioactivity and drug likeness scores of title compounds.

Comp	LogP	TPSA	No. of H-bond acceptors	No. of H-bond donors	No. of rotatable bonds	GPCR ligand	Kinase inhibitor	Protease inhibitor	Enzyme inhibitor	Druglikeness	Log BBB	% HIA
**4a**	5.74	101.73	7	0	6	-0.22	-0.20	-0.09	-0.14	-20.5	3.63647	98.582877
**4b**	3.78	105.13	7	1	4	-0.37	-0.31	-0.40	-0.28	-23.3	0.0222056	94.545202
**4c**	3.82	59.31	4	1	3	-0.26	-0.18	-0.31	-0.21	-11.3	0.135592	95.611921
**4d**	3.02	68.54	5	1	6	-0.39	-0.44	-0.37	-0.24	-17.35	0.104906	95.612031
**4e**	3.03	59.31	4	1	3	-0.34	-0.45	-0.39	-0.23	-31.7	0.766701	95.010104
**4f**	2.15	59.31	4	1	2	-0.49	-0.47	-0.63	-0.32	-11.5	0.628878	94.975635
**4g**	2.16	76.38	5	1	3	-0.26	-0.33	-0.30	-0.12	-10.6	0.620491	95.360538
**4h**	3.98	59.31	4	1	3	-0.24	-0.15	-0.32	-0.21	-10.3	0.191751	95.616308
**4i**	2.71	59.31	4	1	3	-0.69	-0.32	-0.73	-0.38	-11.5	0.657958	95.417777
**4j**	2.98	59.31	4	1	3	-0.41	-0.46	-0.56	-0.29	-10.6	0.208753	95.136674
**4k**	3.54	59.31	4	1	4	-0.33	-0.42	-0.42	-0.22	-13.4	0.212408	95.248380

- Molinspiration - log P- partition coefficient - Druglikeness – yes/no - No. of hydrogen bond acceptors
- PreADMET – ADME – BBB, % HIA - No. of rotatable bonds - Bioactivity score – GPCR ligand
- TPSA- Topological polar surface area - No. of hydrogen bond donors - Kinase inhibitor - Protease inhibitor - Enzyme inhibitor

Figure S1. Diagrammatic representation of 2D modelled binding modes of the lead compounds with the binding domain of Human estrogen receptor alpha protein.

Entry	2D modelled binding modes
**4b**	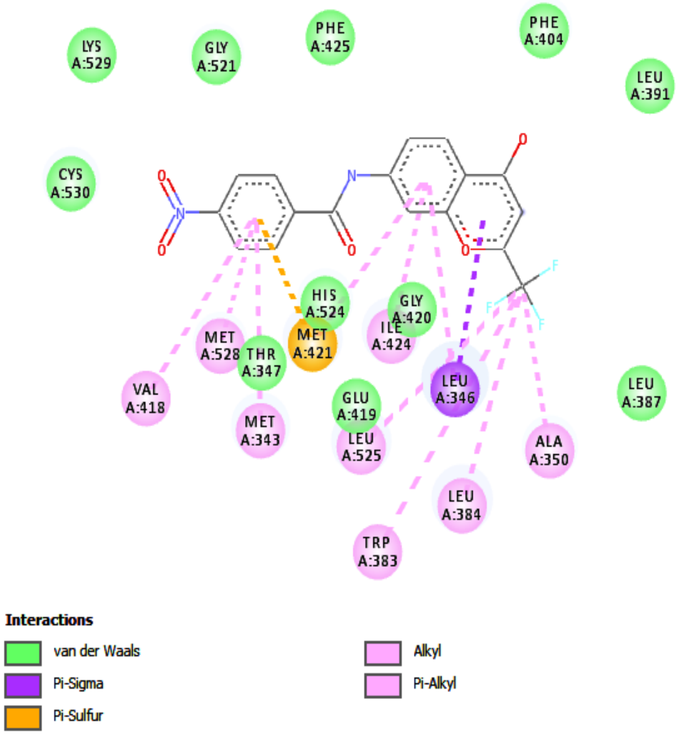
**4c**	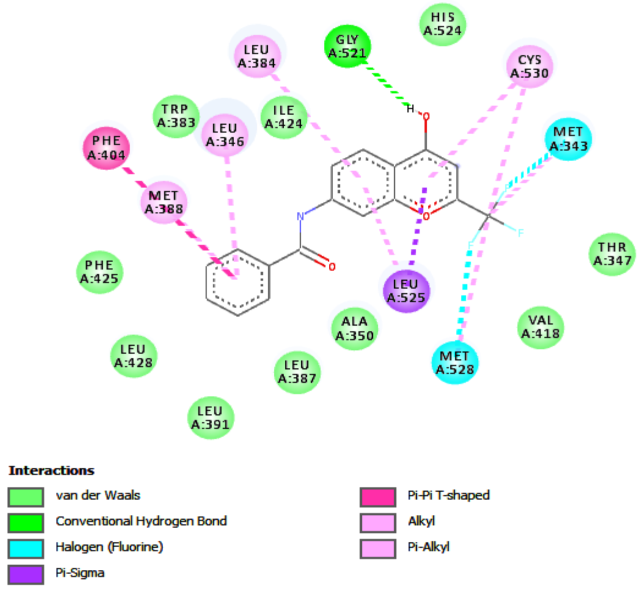

4e

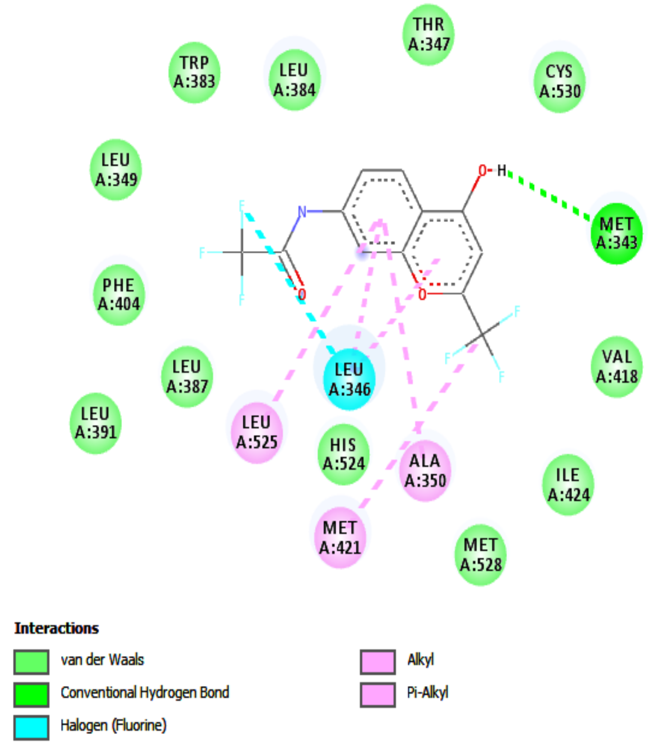
THR A:347
TRP A:383
LEU A:384
CYS A:530
LEU A:349
MET A:343
PHE A:404
LEU A:387
LEU A:346
VAL A:418
LEU A:391
LEU A:525
HIS A:524
ALA A:350
ILE A:424
MET A:421
MET A:528
Interactions
van der Waals
Conventional Hydrogen Bond
Halogen (Fluorine)
Alkyl
Pi-Alkyl

4h

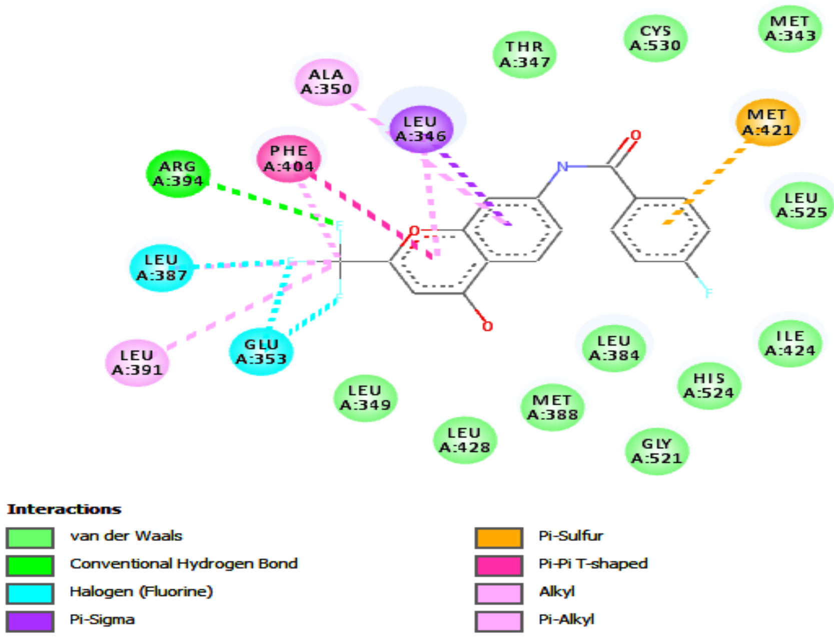
MET A:343
CYS A:530
THR A:347
ALA A:350
LEU A:346
MET A:421
PHE A:404
ARG A:394
LEU A:525
LEU A:387
LEU A:391
GLU A:353
LEU A:384
ILE A:424
HIS A:524
LEU A:349
MET A:388
LEU A:428
GLY A:521
Interactions
van der Waals
Conventional Hydrogen Bond
Halogen (Fluorine)
Pi-Sigma
Pi-Sulfur
Pi-Pi T-shaped
Alkyl
Pi-Alkyl

4k

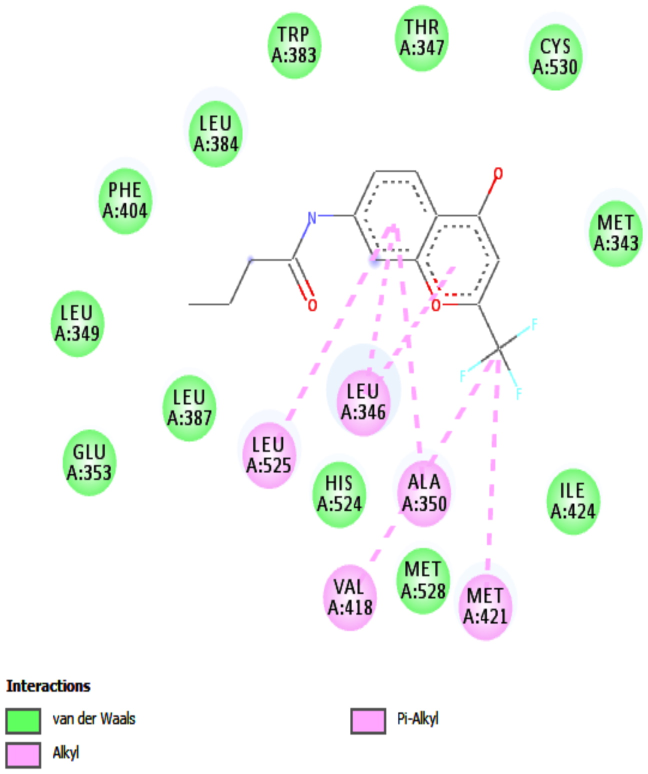
TRP
A:383
THR
A:347
CYS
A:530
LEU
A:384
PHE
A:404
MET
A:343
LEU
A:349
LEU
A:387
LEU
A:346
LEU
A:525
GLU
A:353
HIS
A:524
ALA
A:350
ILE
A:424
VAL
A:418
MET
A:528
MET
A:421
Interactions
van der Waals
Pi-Alkyl
Alkyl

Figure S2. 3D binding domains of synthesized compounds (**4a-k**) and standards against 3MNG protein.

Entry	3D modelled binding modes	Entry	3D modelled binding modes
**4a**	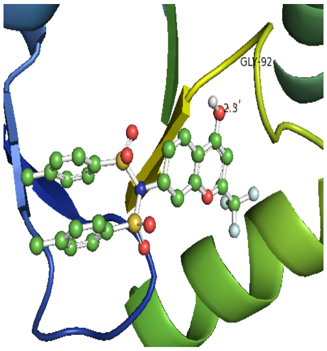	**4g**	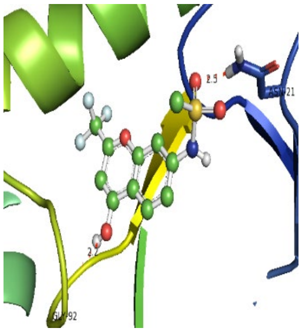
**4b**	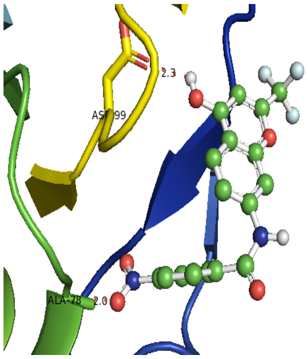	**4h**	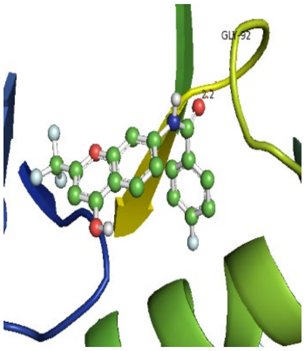
**4c**	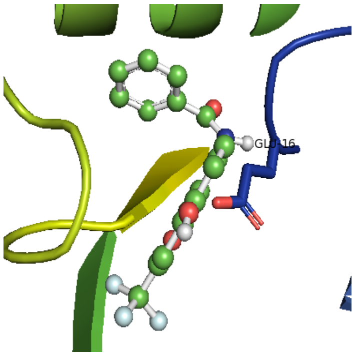	**4i**	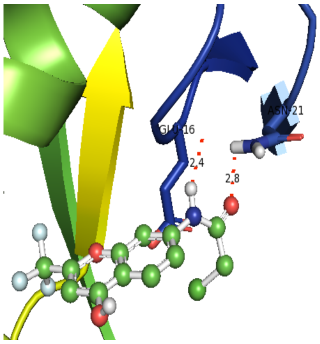

d

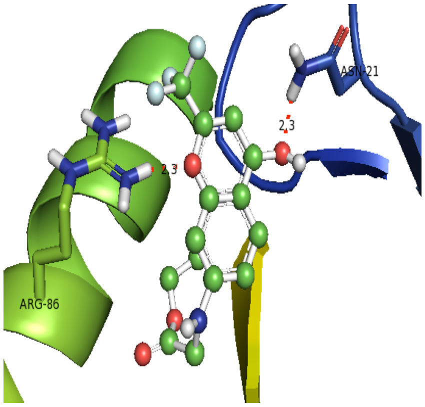

4j

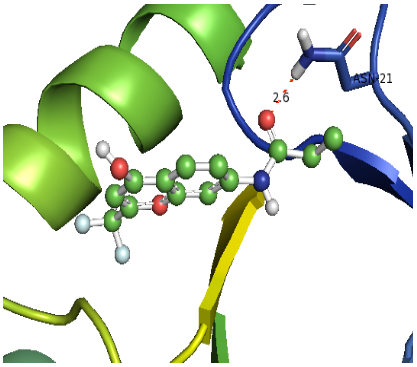

4e

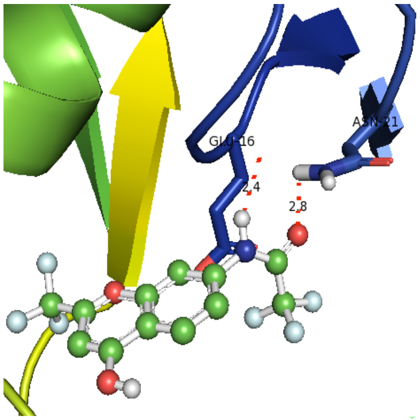

4k

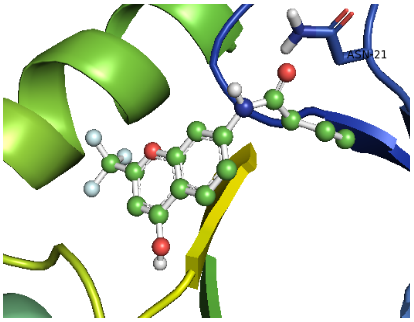

4f

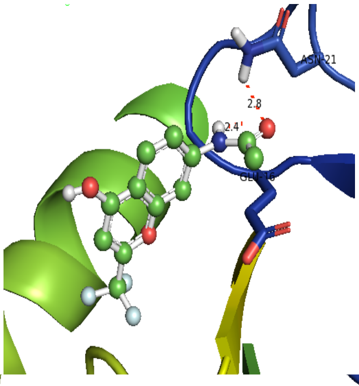

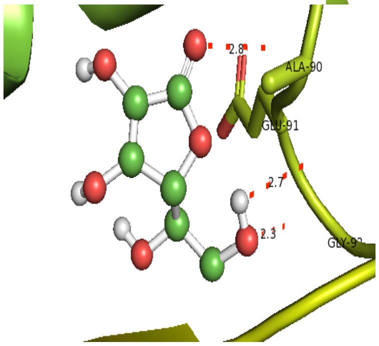

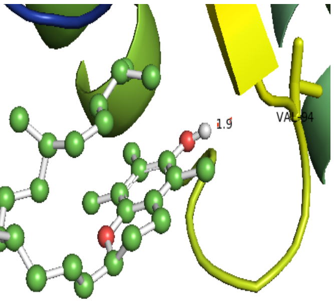

Ascorbic acid **Tocopherol**
